# Integrated analysis of myeloperoxidase in gastric health and cancer: associations with pepsinogen levels, immune regulation, and prognosis in a large healthy population-based and TCGA cohorts

**DOI:** 10.3389/fimmu.2025.1590257

**Published:** 2025-06-06

**Authors:** Junteng Zhou, Qihang Kong, Xiaojing Liu, Yan Huang

**Affiliations:** ^1^ Health Management Center, General Practice Medical Center, West China Hospital, Sichuan University, Chengdu, China; ^2^ Laboratory of Cardiovascular Diseases, Regenerative Medicine Research Center, West China Hospital, Sichuan University, Chengdu, China; ^3^ Department of Cardiology, West China Hospital, Sichuan University, Chengdu, China; ^4^ State Key Laboratory of Respiratory Health and Multimorbidity, West China Hospital, Sichuan University, Chengdu, China; ^5^ Research Laboratory for Prediction and Evaluation of Chronic Diseases in the Elderly, National Clinical Research Center for Geriatric Diseases, Chengdu, China; ^6^ General Practice Research Institute, West China Hospital, Sichuan University, Chengdu, China

**Keywords:** myeloperoxidase, gastric adenocarcinoma, pepsinogen ratio, cross-sectional study, association

## Abstract

**Background:**

Myeloperoxidase (MPO) is a key enzyme involved in immune responses and oxidative stress, yet its roles in gastric physiology and gastric cancer remain incompletely understood. This study comprises two independent analyses: (1) to investigate the association between MPO and gastric mucosal injury markers (pepsinogen I, II, and PGR) in a large healthy population, and (2) to evaluate the prognostic significance and immune-regulatory mechanisms of MPO in gastric adenocarcinoma (GA).

**Methods:**

We analyzed data from 16,943 individuals in a healthy population-based cohort and 375 GA patients from The Cancer Genome Atlas (TCGA). In the healthy cohort, multivariate linear regression was used to evaluate associations between MPO and pepsinogen levels. In the GA cohort, survival analyses (OS, DSS, PFI) were conducted using Kaplan-Meier and Cox regression models. Gene expression analysis, functional enrichment (GO, KEGG, GSEA), and immune infiltration analysis (ssGSEA) were performed to explore MPO-related mechanisms in GA.

**Results:**

In the healthy cohort, MPO was inversely associated with PGR (β = -0.009, P < 0.0001) and PGI (β = -0.057, P < 0.0001). Subgroup and threshold effect analyses revealed non-linear associations and stronger effects among hypertensive individuals, smokers, and alcohol consumers. In the TCGA cohort, high MPO expression was an independent predictor of poor OS (HR = 2.781, P = 0.002) and DSS (HR = 3.667, P < 0.001). Functional analyses showed that MPO was associated with immune-related pathways and increased infiltration of macrophages (R = 0.379, P < 0.001) and dendritic cells (R = 0.377, P < 0.001).

**Conclusion:**

This study highlights the distinct roles of MPO in gastric mucosal injury and gastric cancer. In healthy individuals, MPO is associated with markers of gastric mucosal damage, while in GA patients, MPO serves as a prognostic biomarker linked to immune dysregulation. These findings suggest that MPO may be a potential target for monitoring or intervention in gastric mucosal injury and gastric cancer.

## Introduction

Gastric cancer is a major global health issue that imposes a significant economic burden on cancer patients and society (1). In 2020, gastric cancer ranked among the top three causes of cancer-related deaths in China (2). The majority of gastric cancer cases are gastric adenocarcinomas (3), with treatment primarily including surgery, chemotherapy, radiotherapy, and immunotherapy (4). Due to the high tumor heterogeneity, low early detection rates, and poor prognosis associated with gastric cancer (5), identifying effective prognostic or predictive biomarkers is crucial for improving the management of gastric cancer. The occurrence and progression of gastric cancer result from the combined effects of genetic and environmental factors (6). Previous studies have shown that immune inflammation and related oxidative stress responses are important components of gastric cancer risk (7, 8), but only a few studies have assessed the correlation between the levels of certain oxidative stress biomarkers and gastric cancer. Therefore, there is an urgent need to explore new biomarkers and potential therapeutic targets.

MPO is a heme-containing peroxidase primarily expressed in neutrophils (9), and it serves as one of the sources of reactive oxygen species in immune inflammation processes (10). MPO is part of the innate immune defense against invading pathogens (11). Importantly, MPO is closely associated with tumor development and may play a role in regulating tumor growth, metastasis, and tumor cell migration (12). Studies have found that MPO expression can be detected in the serum of ovarian cancer patients at different stages, while it is undetectable in healthy individuals (13). In a mouse model of pancreatic cancer, MPO deficiency and pharmacological inhibition of MPO, when combined with immune checkpoint therapy, significantly delayed tumor growth (14). Additionally, MPO promotes the migration and invasion of human choriocarcinoma JEG-3 cells (15). However, in breast cancer, MPO-positive cell infiltration is an independent prognostic biomarker (16). These studies suggest a strong connection between MPO and tumors, indicating that MPO may serve as a biomarker for tumor diagnosis, treatment, or prognosis. It is noteworthy that during gastric cancer progression, there is prolonged oxidative stress, accompanied by increased MPO levels (17). However, the function and potential clinical significance of MPO in gastric cancer remain insufficiently explored.

Pepsinogen (PG) is a biomarker commonly used to assess gastric mucosal damage and evaluate gastric health. Pepsinogen I (PGI), Pepsinogen II (PGII), and their ratio (PGR) are closely related to the structure and function of the gastric mucosa, and they can be used to identify populations at increased risk for gastric cancer (18). Studies have shown that serum PG can also help distinguish between Helicobacter pylori-induced and autoimmune atrophic gastritis (19). To the best of our knowledge, PGR and oxidative stress levels are potential diagnostic biomarkers for gastric cancer (20), but research on the relationship between MPO and these PG markers, especially in the context of gastric physiology and pathology, is limited. Therefore, this study investigates the distinct roles of MPO using two independent cohorts. In a large-scale healthy population cohort, we analyzed the relationship between MPO and pepsinogen markers to assess its association with gastric mucosal status. In the TCGA gastric adenocarcinoma cohort, we examined the prognostic value of MPO and explored its potential pathogenic mechanisms through immune microenvironment and functional enrichment analyses, thereby providing a theoretical basis for MPO as a potential biomarker and therapeutic target in gastric mucosal injury and gastric adenocarcinoma.

## Methods

### Overall study design

**Figure 1:**
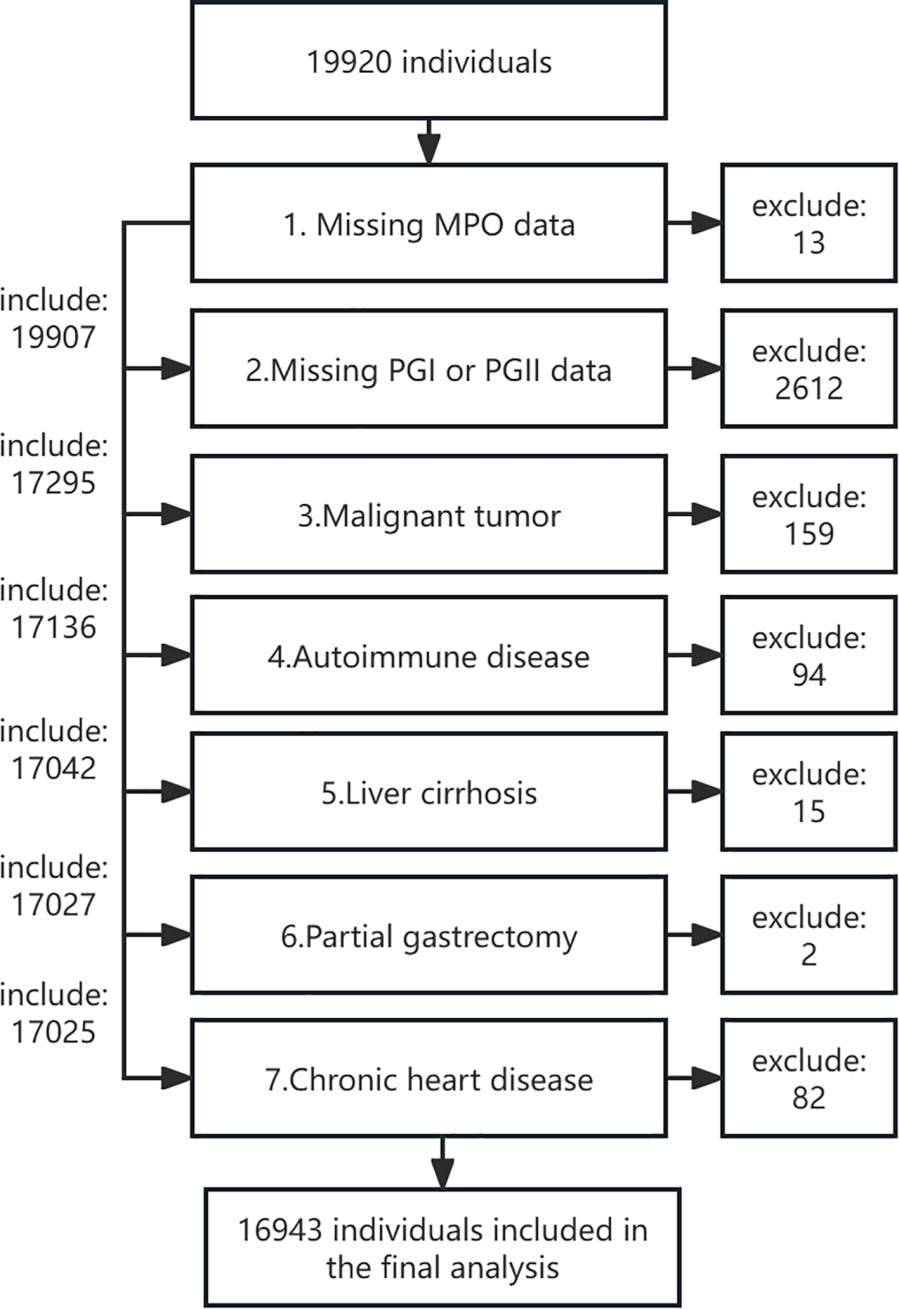
Flow chart of study participants in the healthy population cohort.

This research comprises two independent cohorts with distinct objectives and methodologies. The first and primary cohort is a large, population-based cross-sectional study. It was established between November 2018 and August 2019 among individuals undergoing routine health examinations at the Health Management Center of West China Hospital, Sichuan University. As a tertiary hospital with three subcenters in Sichuan, West China Hospital provides over 60,000 routine physical examinations annually. The study design and participant inclusion/exclusion criteria have been described in detail in our previous publication (21). Briefly, participants were enrolled if they met the following inclusion criteria: (1) voluntarily attended the Health Management Center for health examinations during the study period, (2) were aged over 18 years, and (3) provided informed consent. Exclusion criteria included: (1) missing MPO, PGI, or PGII measurements, (2) history of malignant tumors, (3) autoimmune diseases, (4) liver cirrhosis, (5) partial gastrectomy, and (6) chronic heart disease. Of the initial 19,920 participants, 13 were excluded due to missing MPO data, 2,612 due to missing PGI or PGII data, and 2,625 due to combined missing MPO, PGI, or PGII data. Further exclusions included 159 individuals with malignant tumors, 94 with autoimmune diseases, 15 with liver cirrhosis, 2 with partial gastrectomy, and 82 with chronic heart disease. Ultimately, 16,943 participants were included in the final analysis (**Figure 1**). The study protocol was approved by the Ethics Committee of West China Hospital, Sichuan University (No. 2018-303), and informed consent was obtained from all participants. The study was conducted in accordance with the Declaration of Helsinki.

The second study arm utilized a well-characterized gastric adenocarcinoma cohort from The Cancer Genome Atlas TCGA database, comprising 375 patients with comprehensive molecular profiling and longitudinal clinical follow-up data. This independent analysis specifically examined MPO’s prognostic significance in gastric cancer, employing completely distinct methodologies and endpoints from the healthy population study. The TCGA cohort analysis maintained strict separation from the healthy cohort in terms of study population, biomarkers assessed (RNA expression), and analytical approaches, ensuring no overlap or confounding between these two independent cohorts (**Supplementary Figure S1**).

### Data collection and measurements in the healthy population cohort

Demographic information, including age, sex, smoking status, alcohol consumption, and medical history (e.g., hypertension, diabetes, hyperlipidemia, gout), was collected through standardized questionnaires administered by trained interviewers. Smoking status was categorized as never (smoked fewer than 100 cigarettes), former (not smoked in the past 30 days), or current (smoked in the past 30 days). Alcohol consumption was classified as never (monthly or less), former (abstinence for at least 6 months), or current (at least one alcohol unit per week for more than 6 months). Anthropometric measurements, including height, weight, and blood pressure, were obtained by trained nurses. Body mass index (BMI) was calculated as weight (kg) divided by height squared (m²).

After an overnight fast of at least 8 hours, venous blood samples were collected into 10 mL EDTA tubes from the cubital vein by trained nurses. All blood samples were analyzed in strict accordance with standard laboratory test methods in the clinical laboratory of West China Hospital, which is certified by the China National Accreditation Board. The following parameters were measured: red blood cell count (RBC), white blood cell count (WBC), platelet count, alanine transaminase (ALT), aspartate transaminase (AST), triglycerides (TG), total cholesterol (TC), low-density lipoprotein cholesterol (LDL-C), high-density lipoprotein cholesterol (HDL-C), blood urea nitrogen (BUN), creatinine, uric acid, pepsinogen I (PGI), and pepsinogen II (PGII). PGR was calculated as PGI divided by PGII. The following ELISA kits were used in this study: Gastric PGI ELISA Kit (Catalog No. 601010.01CH) and PGII ELISA Kit (Catalog No. 601020.02CN), all purchased from Biohit, Finland. Each kit has a specification of 96 tests per box. MPO concentrations were measured in plasma samples using a commercial enzyme-linked immunosorbent assay (ELISA) kit (EACHY, Suzhou, China) following the manufacturer’s instructions, as previously described in our earlier publication (21). H. pylori infection was identified using the 14C urea breath test (UBT) (Shenzhen Zhonghe Headway Bio-Sci & Tech Co. Ltd., Shenzhen, Guangdong, China). Participants with a disintegrations per minute (dpm) value ≥ 100 were considered positive for H. pylori infection.

### Molecular and clinical analysis of TCGA gastric adenocarcinoma cohort

RNA sequencing (RNAseq) data and clinical information for gastric adenocarcinoma (STAD) patients were obtained from The Cancer Genome Atlas database (https://portal.gdc.cancer.gov). The data were processed using the STAR pipeline, and transcript per million (TPM) values were extracted for analysis. Normal tissue samples and samples lacking clinical information were excluded, and gene expression data were log2-transformed (log2[TPM + 1]) for normalization. A total of 375 patients with complete clinical and RNAseq data were included in the final analysis. MPO expression levels were quantified using TPM values, and patients were stratified into low and high MPO expression groups based on the median TPM value. Clinical variables, including pathologic T/N/M stage, age, sex, race, histologic grade, pathologic stage, H. pylori infection status, residual tumor status, and survival outcomes (OS, DSS, PFI), were collected for analysis.

### Differential expression analysis

RNA sequencing (RNAseq) data from the TCGA-STAD (Stomach Adenocarcinoma) cohort were processed using the STAR pipeline, and transcript per million (TPM) values were extracted. Differential expression analysis was performed using the DESeq2 package (version 1.36.0) in R (version 4.2.1). MPO (ENSG00000005381.8) expression levels were stratified into low (0-50%) and high (50-100%) expression groups based on the median TPM value, with the low expression group as the reference. The raw counts matrix was analyzed following the standard DESeq2 workflow to identify differentially expressed genes (DEGs). DEGs were defined as genes with an absolute log2 fold change > 1 and an adjusted P-value < 0.05. Data analysis and visualization were performed using the Xiantao Academic Platform (https://www.xiantao.love/).

### Functional enrichment analysis

To explore the biological significance of the identified DEGs, Gene Ontology (GO), Kyoto Encyclopedia of Genes and Genomes (KEGG), and Gene Set Enrichment Analysis (GSEA) were performed using the clusterProfiler package (version 4.4.4) in R. DEGs were annotated and converted to Entrez IDs using the org.Hs.eg.db package. GO enrichment analysis was conducted to identify significant biological processes (BP), cellular components (CC), and molecular functions (MF). KEGG pathway analysis was performed to uncover enriched signaling pathways. Significance was defined as an adjusted P-value < 0.05. Gene Set Enrichment Analysis (GSEA) was performed to evaluate the enrichment of gene sets from the MSigDB Collections (c2.cp.all.v2022.1.Hs.symbols.gmt). DEGs were ranked by log2 fold change, and the analysis was conducted using the default parameters. The reference gene set was obtained from the msigdbr package, and gene IDs were converted using org.Hs.eg.db. Data analysis and visualization were performed using the Xiantao Academic Platform (https://www.xiantao.love/).

### Immune infiltration analysis

Immune cell infiltration levels were estimated using the single-sample gene set enrichment analysis (ssGSEA) algorithm implemented in the GSVA package (version 1.46.0). A panel of 24 immune cell types, including activated dendritic cells (aDC), B cells, CD8 T cells, cytotoxic cells, dendritic cells (DC), eosinophils, immature dendritic cells (iDC), macrophages, mast cells, neutrophils, NK CD56bright cells, NK CD56dim cells, NK cells, plasmacytoid dendritic cells (pDC), T cells, T helper cells, T central memory (Tcm), T effector memory (Tem), T follicular helper (TFH), T gamma delta (Tgd), Th1 cells, Th17 cells, Th2 cells, and regulatory T cells (TReg), were analyzed. Spearman correlation analysis was performed to assess the relationship between MPO expression and immune cell infiltration levels. Results were visualized using the ggplot2 package (version 3.4.4).

### Statistical analysis

Continuous variables are presented as mean ± standard deviation (SD) or median (interquartile range, IQR), depending on their distribution, while categorical variables are reported as counts (percentages). Normality of continuous variables was assessed using the Kolmogorov-Smirnov test and Q-Q plots. Univariate and multivariate linear regression models were used to evaluate the associations between MPO and pepsinogen I, pepsinogen II, and the PGR, with MPO analyzed both as a continuous variable and categorized into quartiles. Cox proportional hazards regression models were employed to assess the associations between MPO expression and survival outcomes (OS, DSS, PFI), with MPO analyzed as a continuous variable. Kaplan-Meier curves and log-rank tests were used to compare survival outcomes between MPO expression groups. Interaction and subgroup analyses explored the effects of potential modifiers (e.g., hypertension, diabetes, smoking status) on the relationship between MPO and PGR. Non-linear relationships were evaluated using restricted cubic spline (RCS) and generalized additive models (GAM), with threshold effect analysis identifying inflection points. Analysis was performed using the Free Statistics software (version 2.0; Beijing FreeClinical Medical Technology Co., Ltd, Beijing, China), with statistical significance determined by two-tailed p-values <0.05.

## Results

### Baseline characteristics across different PGR

The study included 16,943 participants with a mean age of 45.6 ± 12.0 years, of whom 8,111 (47.9%) were female and 8,832 (52.1%) were male. The population was stratified into quartiles based on the pepsinogen ratio (PGI/PGII): Q1 (≤6.09), Q2 (6.10–7.82), Q3 (7.83–9.73), and Q4 (≥9.74) (**Table 1**). Participants in higher quartiles were younger, more likely to be male, and had higher rates of current smoking and alcohol consumption (all P < 0.001). Systolic blood pressure (SBP) showed a decreasing trend across quartiles (P = 0.028). Disease-related indicators varied significantly, with diabetes prevalence increasing across quartiles (P < 0.001) and gout prevalence showing significant differences (P = 0.004). H. pylori infection rates decreased significantly with increasing quartiles (P < 0.001). Laboratory parameters also exhibited significant trends. Liver function markers, including aspartate aminotransferase (AST), showed significant differences (P < 0.001). Lipid profiles varied, with higher quartiles associated with increased triglycerides (TG) and decreased cholesterol, low-density lipoprotein (LDL), and high-density lipoprotein (HDL) levels (all P < 0.001). Blood cell counts, including red blood cells (RBC), white blood cells (WBC), and neutrophils, also showed significant differences across quartiles (all P < 0.001). MPO levels exhibited a significant inverse trend, with lower MPO levels observed in higher quartiles (P < 0.001). To address clinical relevance, we performed additional analyses using the established clinical threshold for atrophic gastritis (PGR ≤3.0). As shown in **Supplementary Table 1**, the 403 participants (2.4%) with PGR ≤3 demonstrated significantly higher MPO levels (median 27.71 vs 25.04 ng/mL, P<0.001) compared to those with PGR >3. This group was older (mean 52.7 vs 45.4 years), had higher systolic blood pressure (mean 119.6 vs 116.2 mmHg), lower lymphocyte counts (30.4% vs 32.1%), and markedly different pepsinogen profiles (PGI: median 45.8 vs 69.7 ng/mL; PGII: median 21.0 vs 8.7 ng/mL; all P<0.001). Notably, H. pylori infection rates were nearly double in the low PGR group (55.9% vs 30.4%, P<0.001). These findings confirm our primary analysis while aligning with clinical diagnostic thresholds.

**Table 1:** Baseline characteristics across PGR quartiles in a healthy population.

	Q1 (<=6.09)	Q2 (6.10-7.82)	Q3 (7.83-9.73)	Q4 (>=9.74)	P-value
No. of participants	4231	4216	4248	4248	
Age (years, mean ± SD)	47.89 ± 11.86	45.82 ± 12.08	44.87 ± 11.96	43.89 ± 11.88	<0.001
Sex, N (%)					<0.001
Female	2320 (54.83%)	2080 (49.34%)	1972 (46.42%)	1739 (40.94%)	
Male	1911 (45.17%)	2136 (50.66%)	2276 (53.58%)	2509 (59.06%)	
BMI (kg/m², mean ± SD)	23.45 ± 3.38	23.51 ± 3.35	23.42 ± 3.39	23.60 ± 3.34	0.068
SBP (mmHg, mean ± SD)	116.86 ± 16.63	116.04 ± 15.98	116.08 ± 15.67	115.94 ± 15.13	0.028
DBP (mmHg, mean ± SD)	72.03 ± 10.52	71.80 ± 10.55	71.83 ± 10.44	71.76 ± 10.46	0.637
Smoking status, N (%)					<0.001
Never	3259 (77.03%)	3106 (73.67%)	3080 (72.50%)	2860 (67.33%)	
Former	207 (4.89%)	196 (4.65%)	159 (3.74%)	188 (4.43%)	
Current	765 (18.08%)	914 (21.68%)	1009 (23.75%)	1200 (28.25%)	
Alcohol status, N (%)					<0.001
Never	2536 (59.94%)	2385 (56.57%)	2366 (55.70%)	2138 (50.33%)	
Former	31 (0.73%)	33 (0.78%)	35 (0.82%)	35 (0.82%)	
Current	1664 (39.33%)	1798 (42.65%)	1847 (43.48%)	2075 (48.85%)	
Hypertension, N (%)					0.658
No	3940 (93.12%)	3950 (93.69%)	3977 (93.62%)	3961 (93.24%)	
Yes	291 (6.88%)	266 (6.31%)	271 (6.38%)	287 (6.76%)	
Diabetes, N (%)					<0.001
No	4143 (97.92%)	4148 (98.39%)	4145 (97.58%)	4110 (96.75%)	
Yes	88 (2.08%)	68 (1.61%)	103 (2.42%)	138 (3.25%)	
Hyperlipidemia, N (%)					0.386
No	4170 (98.56%)	4161 (98.70%)	4175 (98.28%)	4178 (98.35%)	
Yes	61 (1.44%)	55 (1.30%)	73 (1.72%)	70 (1.65%)	
Gout, N (%)					0.004
No	4193 (99.10%)	4170 (98.91%)	4192 (98.68%)	4174 (98.26%)	
Yes	38 (0.90%)	46 (1.09%)	56 (1.32%)	74 (1.74%)	
RBC (*10^12/L, mean ± SD)	4.82 ± 0.54	4.87 ± 0.53	4.89 ± 0.54	4.92 ± 0.54	<0.001
WBC (*10^9/L, mean ± SD)	5.80 ± 1.51	5.78 ± 1.52	5.81 ± 1.54	5.95 ± 1.70	<0.001
Neutrophils (*10^9/L, mean ± SD)	3.49 ± 1.20	3.45 ± 1.18	3.47 ± 1.24	3.55 ± 1.33	<0.001
Lymphocytes (*10^9/L, mean ± SD)	31.76 ± 7.57	32.21 ± 7.39	32.25 ± 7.49	32.11 ± 7.44	0.011
Monocytes (*10^9/L, mean ± SD)	5.98 ± 1.55	6.04 ± 1.51	6.06 ± 1.53	6.00 ± 1.49	0.09
Platelets (*10^9/L, mean ± SD)	202.94 ± 61.27	206.76 ± 60.27	205.57 ± 58.58	208.51 ± 60.66	<0.001
TG (mmol/L, mean ± SD)	1.45 ± 1.11	1.53 ± 1.28	1.59 ± 1.24	1.71 ± 1.46	<0.001
Cholesterol (mmol/L, mean ± SD)	5.02 ± 0.94	4.96 ± 0.95	4.94 ± 0.93	4.87 ± 0.93	<0.001
LDL (mmol/L, mean ± SD)	3.02 ± 0.82	2.97 ± 0.81	2.95 ± 0.79	2.91 ± 0.80	<0.001
HDL (mmol/L, mean ± SD)	1.56 ± 0.42	1.53 ± 0.43	1.51 ± 0.41	1.44 ± 0.40	<0.001
ALT (U/L, median, IQR)	19.00 (14.00-28.00)	20.00 (14.00-30.00)	19.00 (14.00-29.00)	20.00 (14.00-30.00)	0.1
AST (U/L, median, IQR)	21.00 (18.00-26.00)	21.00 (18.00-26.00)	21.00 (17.00-25.00)	20.00 (17.00-25.00)	<0.001
BUN (mmol/L, mean ± SD)	4.89 ± 1.25	4.84 ± 1.22	4.88 ± 1.25	4.89 ± 1.25	0.205
Creatinine (umol/L, mean ± SD)	68.22 ± 16.10	69.59 ± 14.84	70.26 ± 15.90	72.27 ± 16.78	<0.001
MPO (ng/mL, median, IQR)	27.07 (20.21-38.08)	25.83 (19.34-35.87)	24.14 (17.39-33.94)	23.07 (16.11-33.63)	<0.001
Pepsinogen I (ng/ml, median, IQR)	70.30 (51.72-95.24)	65.64 (52.01-86.35)	67.92 (55.09-86.74)	73.05 (59.27-92.33)	<0.001
Pepsinogen II (ng/ml, median, IQR)	14.99 (10.71-21.16)	9.48 (7.43-12.46)	7.77 (6.30-10.03)	6.27 (5.03-7.99)	<0.001
PGR	4.66 ± 1.16	6.96 ± 0.50	8.72 ± 0.54	11.87 ± 2.07	<0.001
H. pylori infection, N (%)					<0.001
No	1675 (44.07%)	2593 (68.02%)	3033 (79.42%)	3200 (84.72%)	
Yes	2126 (55.93%)	1219 (31.98%)	786 (20.58%)	577 (15.28%)	

### Association between MPO and pepsinogen levels: multivariate analysis

To investigate the relationship between MPO and pepsinogen levels, we employed multiple regression models with three adjustment approaches: non-adjusted, adjusted for sex and age (Model I), and adjusted for sex, age, BMI, systolic blood pressure, smoking status, alcohol consumption, diabetes, gout, blood cell counts, lipid profiles, and liver/kidney function markers (Model II) (**Table 2**).

**Table 2:** Multivariate analysis of MPO’s association with PGR, pepsinogen I, and pepsinogen II.

	Non-adjusted (β,95% CI, P value)	Adjust model I (β,95% CI, P value)	Adjust model II (β,95% CI, P value)
PGR			
MPO as Continuous	-0.008 (-0.010, -0.007) <0.0001	-0.009 (-0.011, -0.008) <0.0001	-0.009 (-0.010, -0.007) <0.0001
MPO as quartile			
Q1	Ref	Ref	Ref
Q2	-0.594 (-0.717, -0.471) <0.00001	-0.597 (-0.718, -0.475) <0.0001	-0.661 (-0.782, -0.541) <0.0001
Q3	-0.889 (-1.012, -0.766) <0.0001	-0.903 (-1.024, -0.782) <0.0001	-0.923 (-1.045, -0.802) <0.0001
Q4	-0.847 (-0.970, -0.724) <0.0001	-0.923 (-1.045, -0.801) <0.0001	-0.899 (-1.024, -0.774) <0.0001
P for trend	<0.0001	<0.0001	<0.0001
Pepsinogen I			
MPO as Continuous	-0.056 (-0.076, -0.037) <0.0001	-0.034 (-0.053, -0.015) 0.00041	-0.057 (-0.076, -0.038) <0.0001
MPO as quartile			
Q1	Ref	Ref	Ref
Q2	-1.854 (-3.312, -0.396) 0.01271	-1.360 (-2.760, 0.041) 0.05703	-1.051 (-2.419, 0.318) 0.13247
Q3	-4.777 (-6.235, -3.319) <0.0001	-3.711 (-5.112, -2.309) <0.0001	-3.166 (-4.546, -1.786) <0.0001
Q4	-4.219 (-5.677, -2.761) <0.0001	-2.165 (-3.570, -0.759) 0.00254	-3.515 (-4.931, -2.099) <0.0001
P for trend	<0.0001	<0.0001	<0.0001
Pepsinogen II			
MPO as Continuous	0.004 (0.000, 0.009) 0.04154	0.009 (0.005, 0.013) 0.00003	0.003 (-0.001, 0.007) 0.09634
MPO as quartile			
Q1	Ref	Ref	Ref
Q2	0.526 (0.208, 0.844) 0.00119	0.593 (0.283, 0.903) 0.00018	0.730 (0.433, 1.027) <0.0001
Q3	0.580 (0.262, 0.898) 0.00035	0.742 (0.432, 1.052) <0.0001	0.836 (0.537, 1.136) <0.0001
Q4	0.764 (0.446, 1.082) <0.0001	1.166 (0.855, 1.476) <0.0001	0.890 (0.583, 1.197) <0.0001
P for trend	<0.0001	<0.0001	<0.0001

Non-adjusted model: No adjustments.

Adjusted model I: Adjusted for sex and age.

Adjusted model II: Adjusted for sex, age, BMI, systolic blood pressure, smoking status, alcohol consumption, diabetes, gout, H. pylori infection, red blood cell count, white blood cell count, neutrophil count, lymphocyte count, platelet count, triglycerides, cholesterol, low-density lipoprotein, high-density lipoprotein, aspartate aminotransferase, and creatinine.

β-values: Unstandardized regression coefficients. 95% CI: 95% confidence interval.

In the non-adjusted model, MPO as a continuous variable was significantly associated with a decrease in the PGR (β = -0.008, 95% CI: -0.01 to -0.007, P < 0.0001) and pepsinogen I (β = -0.06, 95% CI: -0.08 to -0.04, P < 0.0001), as well as an increase in pepsinogen II (β = 0.004, 95% CI: 0.00 to 0.01, P = 0.0415). However, the association between MPO and pepsinogen II as a continuous variable lost significance in Model II (β = 0.003, 95% CI: -0.001 to 0.007, P = 0.0963).

When MPO was divided into quartiles, compared with Q1, the adjusted beta coefficients (β) for the PGR in Q2–Q4 were -0.66 (95% CI: -0.78 to -0.54), -0.92 (95% CI: -1.05 to -0.80), and -0.90 (95% CI: -1.02 to -0.77), respectively, with P for trend < 0.0001. Similarly, for pepsinogen I, the adjusted β values in Q2–Q4 were -1.05 (95% CI: -2.42 to 0.32), -3.17 (95% CI: -4.55 to -1.79), and -3.52 (95% CI: -4.93 to -2.10), respectively, with P for trend < 0.0001. For pepsinogen II, the adjusted β values in Q2–Q4 were 0.73 (95% CI: 0.43 to 1.03), 0.84 (95% CI: 0.54 to 1.14), and 0.89 (95% CI: 0.58 to 1.20), respectively, with P for trend < 0.0001.

In addition, multivariable analyses confirmed the correlation between MPO and PGR ≤3 status across modeling approaches (**Supplementary Table 2**). For continuous MPO, each unit increase was associated with higher risk (Non-adjusted: HR=1.003, 95%CI 1.000–1.006, P=0.048; Model II: HR=1.003, 1.000–1.006, P=0.033). Quartile analysis revealed a striking dose-response relationship (P for trend < 0.00001), with the highest MPO quartile (Q4) showing nearly doubled risk versus Q1 (Non-adjusted HR=1.758, 1.312–2.357, P=0.0002; Model II HR=1.989, 1.448–2.731, P=0.00002). These results demonstrate that elevated MPO levels are consistently associated with reduced pepsinogen ratios (PGR ≤ 3). To address potential confounding by H. pylori infection, we stratified analyses by H. pylori status (**Supplementary Table 3**). The inverse association between MPO and PGR persisted in both H. pylori-negative (β = −0.009, 95% CI: −0.011 to −0.007, P < 0.0001) and H. pylori-positive subgroups (β = −0.008, −0.011 to −0.005, P < 0.0001) in fully adjusted models. Consistent trends were observed when MPO was analyzed as quartiles (P for trend < 0.0001 in both subgroups), with effect sizes mirroring the overall cohort (**Table 2**).

### Subgroup analysis of the association between MPO and PGR

**Figure 2:**
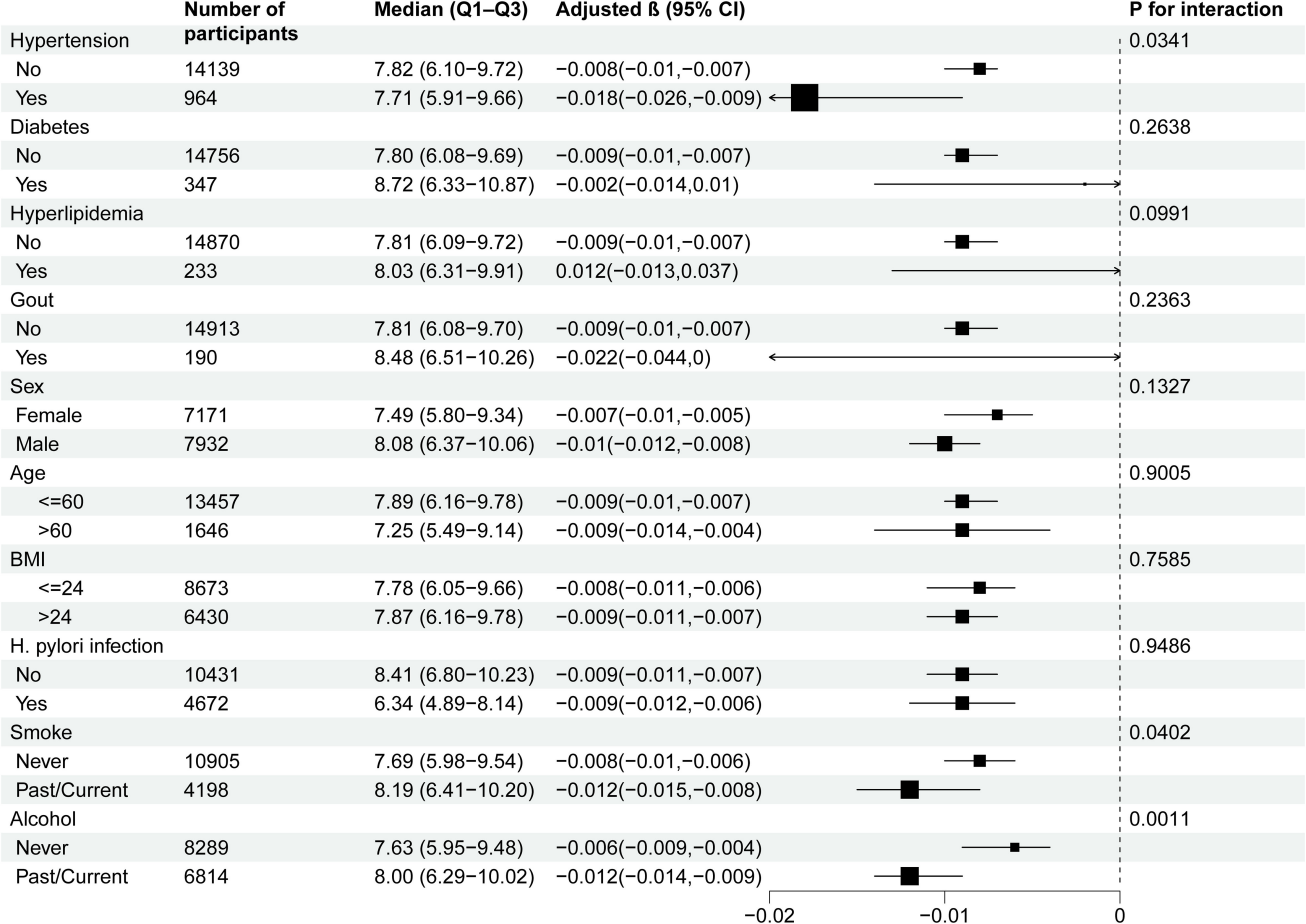
Subgroup analyses of the effect of MPO on PGR. Adjustment for: Sex, age, BMI, systolic blood pressure, smoking status, alcohol consumption, diabetes, gout, H. pylori infection, red blood cell count, white blood cell count, neutrophil count, lymphocyte count, platelet count, triglycerides, cholesterol, low-density lipoprotein, high-density lipoprotein, aspartate aminotransferase, and creatinine, except the stratification variable in each case.

To further assess potential moderating factors on the association between MPO and PGR, we conducted subgroup and interaction analyses (**Table 3** and **Figure 2**). Significant interactions were observed for hypertension (P for interaction = 0.0341), smoking status (P for interaction = 0.0402), and alcohol consumption (P for interaction = 0.0011). Specifically, the inverse association between MPO and PGR was stronger in participants with hypertension (β = -0.018, 95% CI: -0.026 to -0.009) compared to those without hypertension (β = -0.008, 95% CI: -0.01 to -0.007). Similarly, the association was more pronounced in past/current smokers (β = -0.012, 95% CI: -0.015 to -0.008) compared to never smokers (β = -0.008, 95% CI: -0.01 to -0.006) and in past/current alcohol consumers (β = -0.012, 95% CI: -0.014 to -0.009) compared to never drinkers (β = -0.006, 95% CI: -0.009 to -0.004). No significant interactions were observed for diabetes, hyperlipidemia, gout, sex, age, BMI) and H. pylori infection.

**Table 3:** Effect size of MPO on PGR in prespecified and exploratory subgroups.

	No of participants	Median (Q1–Q3)	Adjusted β (95% CI)	P for interaction
Hypertension				0.0341
No	14139	7.82 (6.10-9.72)	-0.008 (-0.01,-0.007)	
Yes	964	7.71 (5.91-9.66)	-0.018 (-0.026,-0.009)	
Diabetes				0.2638
No	14756	7.80 (6.08-9.69)	-0.009 (-0.01,-0.007)	
Yes	347	8.72 (6.33-10.87)	-0.002 (-0.014,0.01)	
Hyperlipidemia				0.0991
No	14870	7.81 (6.09-9.72)	-0.009 (-0.01,-0.007)	
Yes	233	8.03 (6.31-9.91)	0.012 (-0.013,0.037)	
Gout				0.2363
No	14913	7.81 (6.08-9.70)	-0.009 (-0.01,-0.007)	
Yes	190	8.48 (6.51-10.26)	-0.022 (-0.044,0)	
Sex				0.1327
Female	7171	7.49 (5.80-9.34)	-0.007 (-0.01,-0.005)	
Male	7932	8.08 (6.37-10.06)	-0.01 (-0.012,-0.008)	
Age				0.901
<=60	13457	7.89 (6.16-9.78)	-0.009 (-0.01,-0.007)	
>60	1646	7.25 (5.49-9.14)	-0.009 (-0.014,-0.004)	
BMI				0.7585
<=24	8673	7.78 (6.05-9.66)	-0.008 (-0.011,-0.006)	
>24	6430	7.87 (6.16-9.78)	-0.009 (-0.011,-0.007)	
H. pylori infection				0.9486
No	10431	8.41 (6.80-10.23)	-0.009 (-0.011,-0.007)	
Yes	4672	6.34 (4.89-8.14)	-0.009 (-0.012,-0.006)	
Smoke				0.0402
Never	10905	7.69 (5.98-9.54)	-0.008 (-0.01,-0.006)	
Past/Current	4198	8.19 (6.41-10.20)	-0.012 (-0.015,-0.008)	
Alcohol				0.0011
Never	8289	7.63 (5.95-9.48)	-0.006 (-0.009,-0.004)	
Past/Current	6814	8.00 (6.29-10.02)	-0.012 (-0.014,-0.009)	

Adjusted for sex, age, BMI, systolic blood pressure, smoking status, alcohol consumption, diabetes, gout, H. pylori infection, red blood cell count, white blood cell count, neutrophil count, lymphocyte count, platelet count, triglycerides, cholesterol, low-density lipoprotein, high-density lipoprotein, aspartate aminotransferase, and creatinine except interaction factor.

### Threshold effects of MPO on pepsinogen levels

**Figure 3:**
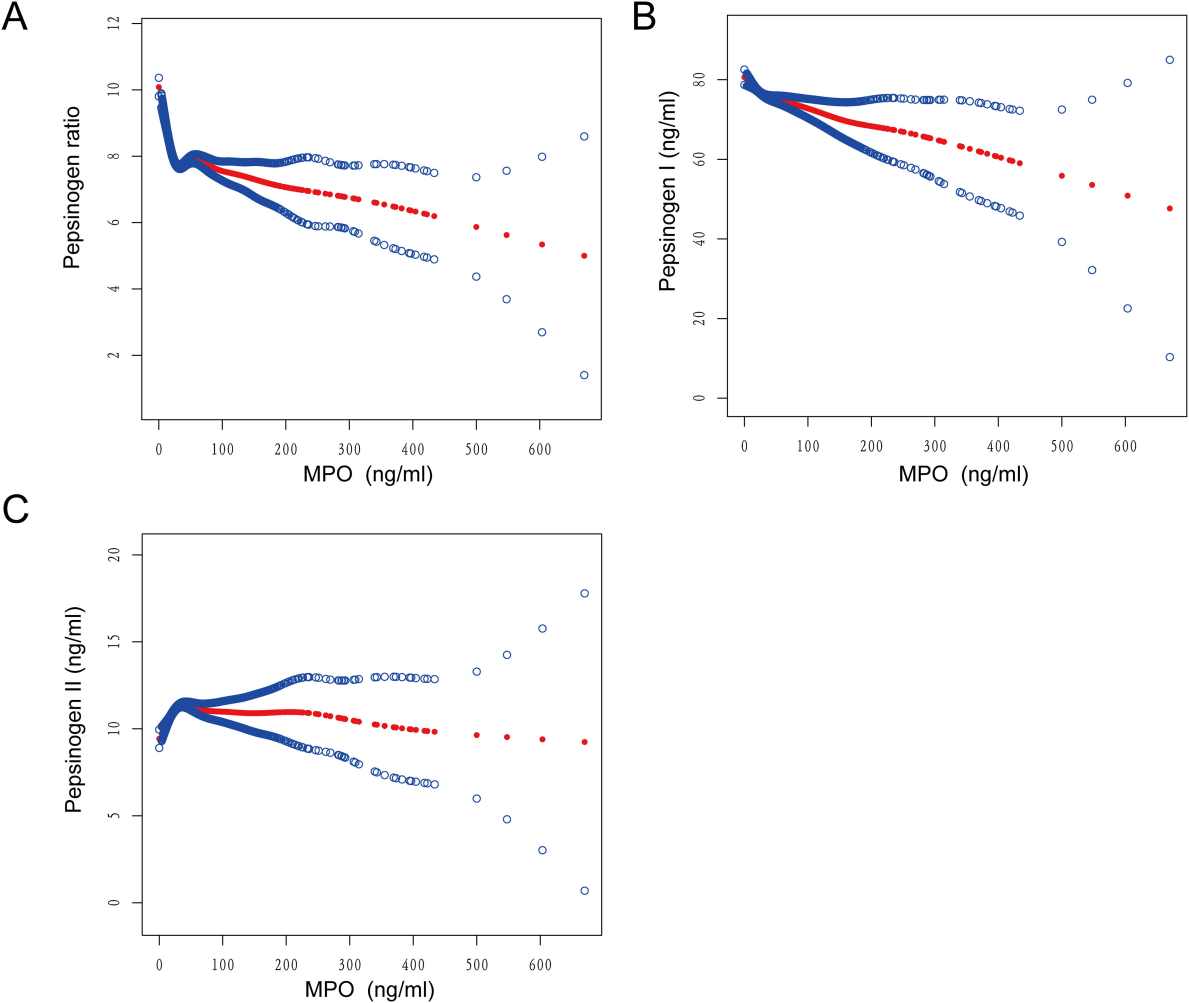
Smooth curve fitting was used to assess the relationship between MPO and **(A)** PGR, **(B)** pepsinogen I, and **(C)** pepsinogen II.

To investigate the linearity of the relationship between MPO and pepsinogen levels, we employed smooth curve fitting and segmented regression models (**Figures 3A-C**). Based on the restricted cubic spline (RCS) curves and GAM model, we identified an inflection point at MPO = 24. After adjusting for confounding factors, we observed nonlinear associations between MPO and pepsinogen levels (**Table 4**).

**Table 4:** Nonlinear concentration-effect relationship of MPO with PGR, pepsinogen I, and pepsinogen II.

	PGR	Pepsinogen I	Pepsinogen II
Model I (Linear Effect)			
	-0.009 (-0.010, -0.007) <0.0001	-0.057 (-0.076, -0.038) <0.0001	0.003 (-0.001, 0.007) 0.0963
Model II (Linear Effect)			
Knockout point (K)	24	24	24
< K-segment effect value	-0.089 (-0.098, -0.079) <0.0001	-0.244 (-0.356, -0.131) <0.0001	0.091 (0.067, 0.116) <0.0001
> K-segment effect value	-0.003 (-0.004, -0.001) 0.0032	-0.043 (-0.063, -0.023) <0.0001	-0.003 (-0.008, 0.001) 0.1642
Difference Between Effect 2 and 1	0.086 (0.075, 0.096) <0.0001	0.201 (0.082, 0.320) 0.0009	-0.094 (-0.120, -0.069) <0.0001
Predicted Value at Threshold	7.812 (7.749, 7.875)	75.793 (75.051, 76.536)	11.189 (11.025, 11.353)
Log-Likelihood Ratio Test	<0.001	<0.001	<0.001

Model I: Linear effect of MPO on pepsinogen levels.

Model II: Threshold effect of MPO on pepsinogen levels, with an inflection point (K) identified at MPO = 24.

Adjustment for: Sex, age, BMI, systolic blood pressure, smoking status, alcohol consumption, diabetes, gout, H. pylori infection, red blood cell count, white blood cell count, neutrophil count, lymphocyte count, platelet count, triglycerides, cholesterol, low-density lipoprotein, high-density lipoprotein, aspartate aminotransferase, and creatinine.

For the PGR, below the inflection point (MPO < 24), each unit increase in MPO was associated with a significant decrease (β = -0.089, 95% CI: -0.098 to -0.079, P < 0.0001). Above the inflection point (MPO ≥ 24), the effect was attenuated but remained significant (β = -0.003, 95% CI: -0.004 to -0.001, P = 0.0032). The difference between the two segments was statistically significant (P < 0.0001), and the log-likelihood ratio test confirmed the nonlinear relationship (P < 0.001).

For pepsinogen I, below the inflection point (MPO < 24), each unit increase in MPO was associated with a significant decrease (β = -0.244, 95% CI: -0.356 to -0.131, P < 0.0001). Above the inflection point (MPO ≥ 24), the effect remained significant but was smaller (β = -0.043, 95% CI: -0.063 to -0.023, P < 0.0001).

For pepsinogen II, below the inflection point, each unit increase in MPO was associated with a significant increase (β = 0.091, 95% CI: 0.067 to 0.116, P < 0.0001). Above the inflection point (MPO ≥ 24), the effect was no longer significant (β = -0.003, 95% CI: -0.008 to 0.001, P = 0.1642).

### Clinicopathological characteristics by MPO expression in TCGA gastric adenocarcinoma cohort

We analyzed data from 375 gastric adenocarcinoma patients in the TCGA cohort, stratifying them into low (n = 187) and high (n = 188) MPO expression groups (**Table 5**). No significant differences were observed for pathologic T stage (P = 0.965), N stage (P = 0.064), M stage (P = 0.870), age (P = 0.963), sex (P = 0.122), race (P = 0.264), histologic grade (P = 0.070), pathologic stage (P = 0.529), H. pylori infection (P = 0.931), or residual tumor status (P = 0.836). Importantly, high MPO expression was significantly associated with worse survival outcomes: Overall survival (OS): Higher mortality in the high MPO group (46.3% vs. 32.1%, P = 0.005). Disease-specific survival (DSS): More disease-specific events in the high MPO group (31.5% vs. 19.9%, P = 0.013). Progression-free interval (PFI): More progression events in the high MPO group (40.4% vs. 25.7%, P = 0.002).

**Table 5:** Baseline clinical characteristics across different MPO expression groups.

	Low expression of MPO	High expression of MPO	P-value
No. of participants	187	188	
Pathologic T stage, n (%)			0.965
T1&T2	49 (26.8%)	50 (27.2%)	
T3	85 (46.4%)	83 (45.1%)	
T4	49 (26.8%)	51 (27.7%)	
Pathologic N stage, n (%)			0.064
N0	66 (37.3%)	45 (25%)	
N1	40 (22.6%)	57 (31.7%)	
N2	36 (20.3%)	39 (21.7%)	
N3	35 (19.8%)	39 (21.7%)	
Pathologic M stage, n (%)			0.870
M0	164 (93.2%)	166 (92.7%)	
M1	12 (6.8%)	13 (7.3%)	
Age, n (%)			0.963
<= 65	82 (44.3%)	82 (44.1%)	
> 65	103 (55.7%)	104 (55.9%)	
Sex, n (%)			0.122
Female	74 (39.6%)	60 (31.9%)	
Male	113 (60.4%)	128 (68.1%)	
Race, n (%)			0.264
Asian&Black or African American	46 (29.1%)	39 (23.6%)	
White	112 (70.9%)	126 (76.4%)	
Histologic grade, n (%)			0.070
G1&G2	82 (44.8%)	65 (35.5%)	
G3	101 (55.2%)	118 (64.5%)	
Pathologic stage, n (%)			0.529
Stage I	30 (17%)	23 (13.1%)	
Stage II	56 (31.8%)	55 (31.2%)	
Stage III&Stage IV	90 (51.1%)	98 (55.7%)	
H pylori infection, n (%)			0.931
No	79 (88.8%)	66 (89.2%)	
Yes	10 (11.2%)	8 (10.8%)	
Residual tumor, n (%)			0.836
R0	148 (90.2%)	150 (90.9%)	
R1&R2	16 (9.8%)	15 (9.1%)	
H pylori infection, n (%)			0.931
No	79 (88.8%)	66 (89.2%)	
Yes	10 (11.2%)	8 (10.8%)	
OS event, n (%)			0.005
Alive	127 (67.9%)	101 (53.7%)	
Dead	60 (32.1%)	87 (46.3%)	
DSS event, n (%)			0.013
No	141 (80.1%)	122 (68.5%)	
Yes	35 (19.9%)	56 (31.5%)	
PFI event, n (%)			0.002
No	139 (74.3%)	112 (59.6%)	
Yes	48 (25.7%)	76 (40.4%)	

### Survival analysis and prognostic significance of MPO

**Figure 4:**
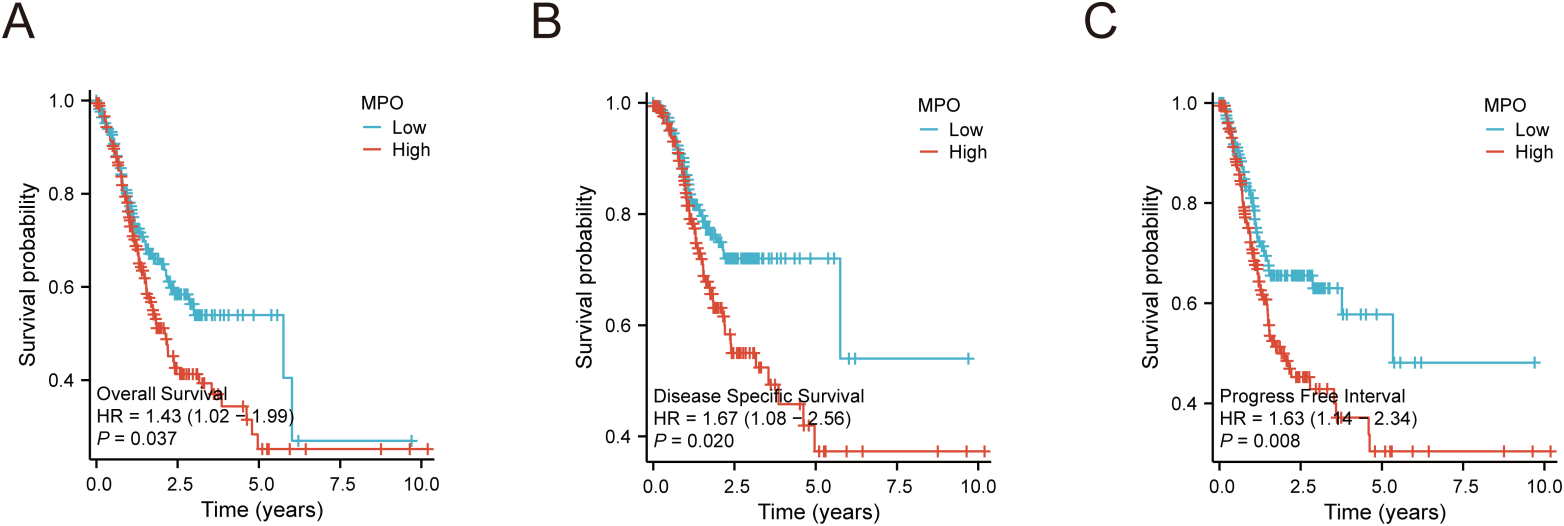
Kaplan-Meier survival curves for MPO expression in gastric adenocarcinoma. **(A)** Overall survival (OS) by MPO expression, **(B)** Disease-specific survival (DSS) by MPO expression, **(C)** Progression-free interval (PFI) by MPO expression.

Kaplan-Meier survival analysis revealed that high MPO expression was significantly associated with worse survival outcomes compared to low MPO expression, including poorer overall survival (OS: HR = 1.43, 95% CI: 1.02–1.99, P = 0.037), disease-specific survival (DSS: HR = 1.67, 95% CI: 1.08–2.56, P = 0.020), and progression-free interval (PFI: HR = 1.63, 95% CI: 1.14–2.34, P = 0.008) (**Figures 4A-C**).

**Figure 5:**
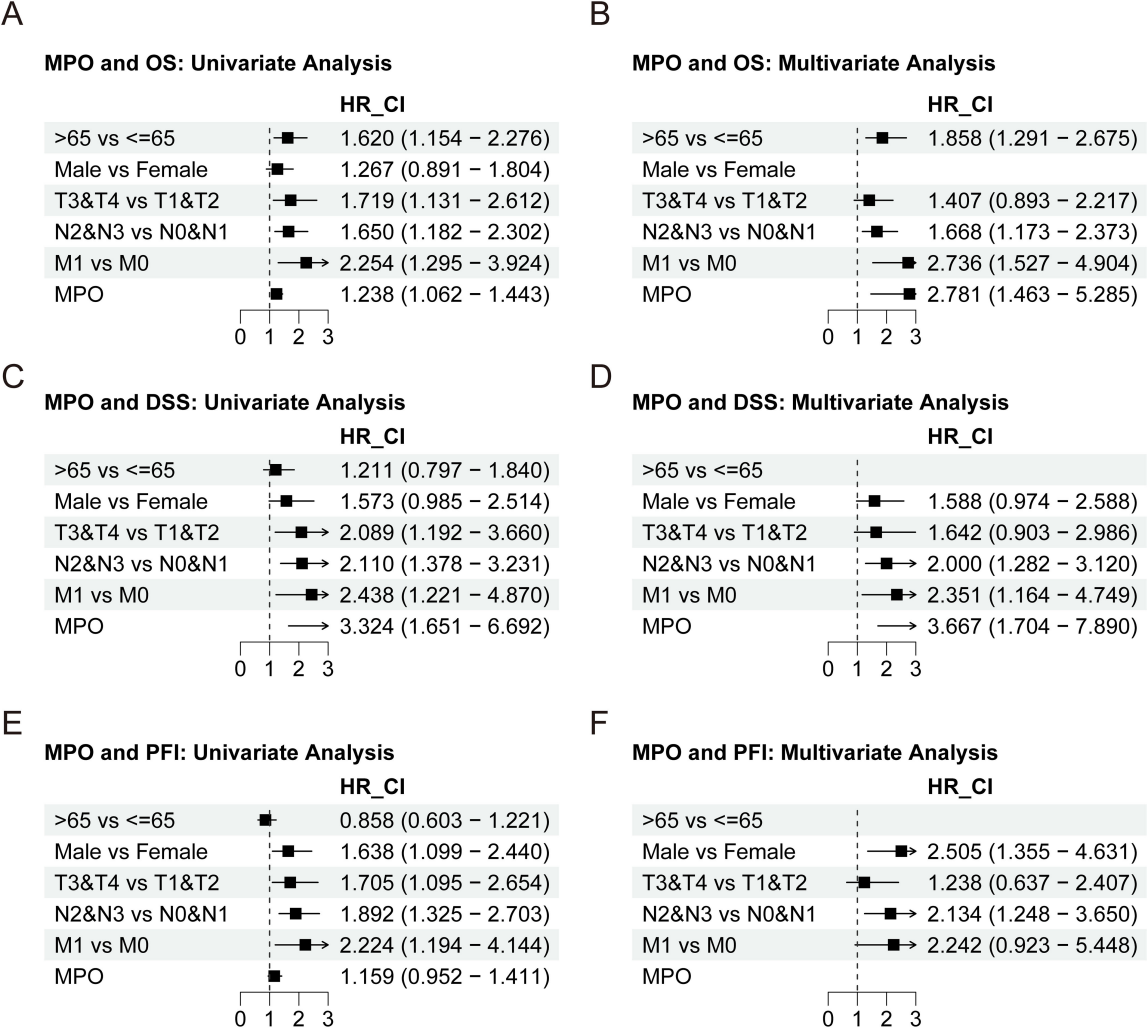
Univariate and multivariate Cox regression analyses of MPO expression. **(A)** Univariate analysis for OS, **(B)** Multivariate analysis for OS, **(C)** Univariate analysis for DSS, **(D)** Multivariate analysis for DSS, **(E)** Univariate analysis for PFI, **(F)** Multivariate analysis for PFI.

To further investigate the prognostic value of MPO, univariate and multivariate analyses were performed. Univariate analysis demonstrated that MPO expression was significantly associated with OS (HR = 1.238, 95% CI: 1.062–1.443, P = 0.006) and DSS (HR = 3.324, 95% CI: 1.651–6.692, P < 0.001), with a trend toward association with PFI (HR = 1.159, 95% CI: 0.952–1.411, P = 0.142). Multivariate analysis confirmed MPO as an independent prognostic factor for OS (HR = 2.781, 95% CI: 1.463–5.285, P = 0.002) and DSS (HR = 3.667, 95% CI: 1.704–7.890, P < 0.001), although its association with PFI did not reach statistical significance (**Figures 5A-F**, **Supplementary Tables 4-6**).

### MPO-associated differential gene expression, functional enrichment, and immune regulation in gastric adenocarcinoma

**Figure 6:**
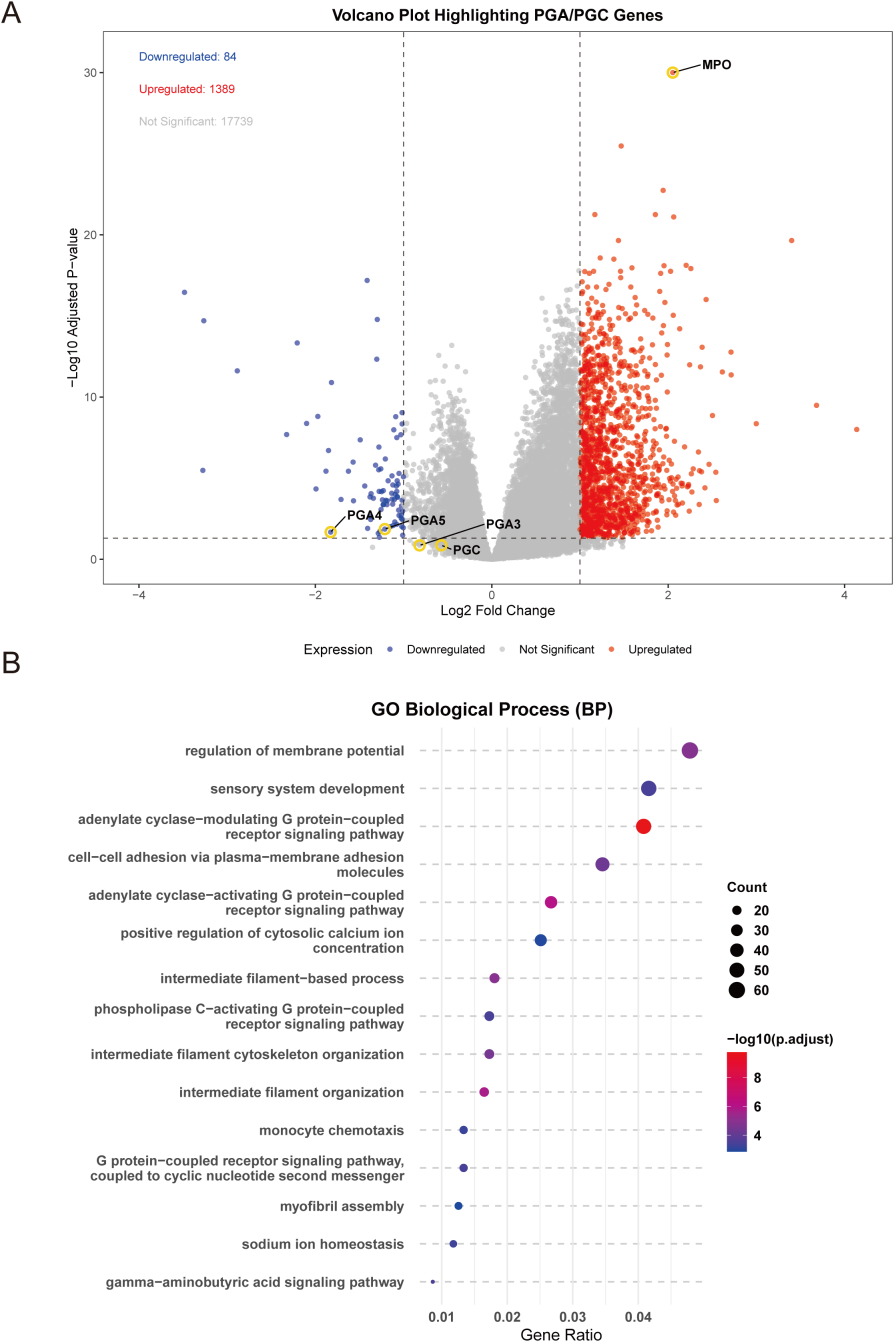
Differential gene expression and functional enrichment analysis of MPO in gastric adenocarcinoma. **(A)** Volcano plot showing differentially expressed genes (DEGs) between high and low MPO expression groups, **(B)** Bubble plot of enriched biological processes (BP) based on GO analysis.

To explore the biological significance of MPO expression in gastric adenocarcinoma, we performed differential gene expression analysis between high and low MPO expression groups. A total of 1,473 differentially expressed genes (DEGs) were identified, including 1,389 upregulated and 84 downregulated genes (|logFC| > 1, adjusted P < 0.05) (**Figure 6A**). Notably, among the downregulated genes, we observed significant suppression of PGI-associated genes (PG4, PG5; FDR < 0.05), while PGII-associated gene (PGC) expression remained unchanged. This selective downregulation pattern in gastric cancer parallels our findings in healthy cohorts, where elevated MPO was associated with reduced PGR (primarily driven by PGI suppression). Further supporting this potential conserved relationship, analysis of the GSE54129 dataset (containing mixed normal and tumor samples) revealed a significant negative correlation between MPO levels and pepsinogen A (PGA) expression (r = -0.187, p < 0.05; **Supplementary Figure S2**).

Differential gene enrichment analysis between high and low MPO expression groups in TCGA gastric adenocarcinoma revealed that these DEGs were significantly associated with key biological processes, cellular components, molecular functions, and signaling pathways. Specifically, DEGs were enriched in G protein-coupled receptor signaling (e.g., adenylate cyclase-modulating and -activating pathways), intermediate filament organization, and regulation of membrane potential (BP) (**Figure 6B**); extracellular matrix, sarcomere, myofibril, and ion channel complexes (CC) (**Supplementary Figure S3**); receptor ligand activity, hormone activity, and extracellular matrix structural constituents (MF) (**Supplementary Figure S4**); and pathways such as cytoskeleton regulation in muscle cells, nicotine addiction, cytokine-cytokine receptor interaction, and cAMP signaling (KEGG) (**Supplementary Figure S5**).

**Figure 7:**
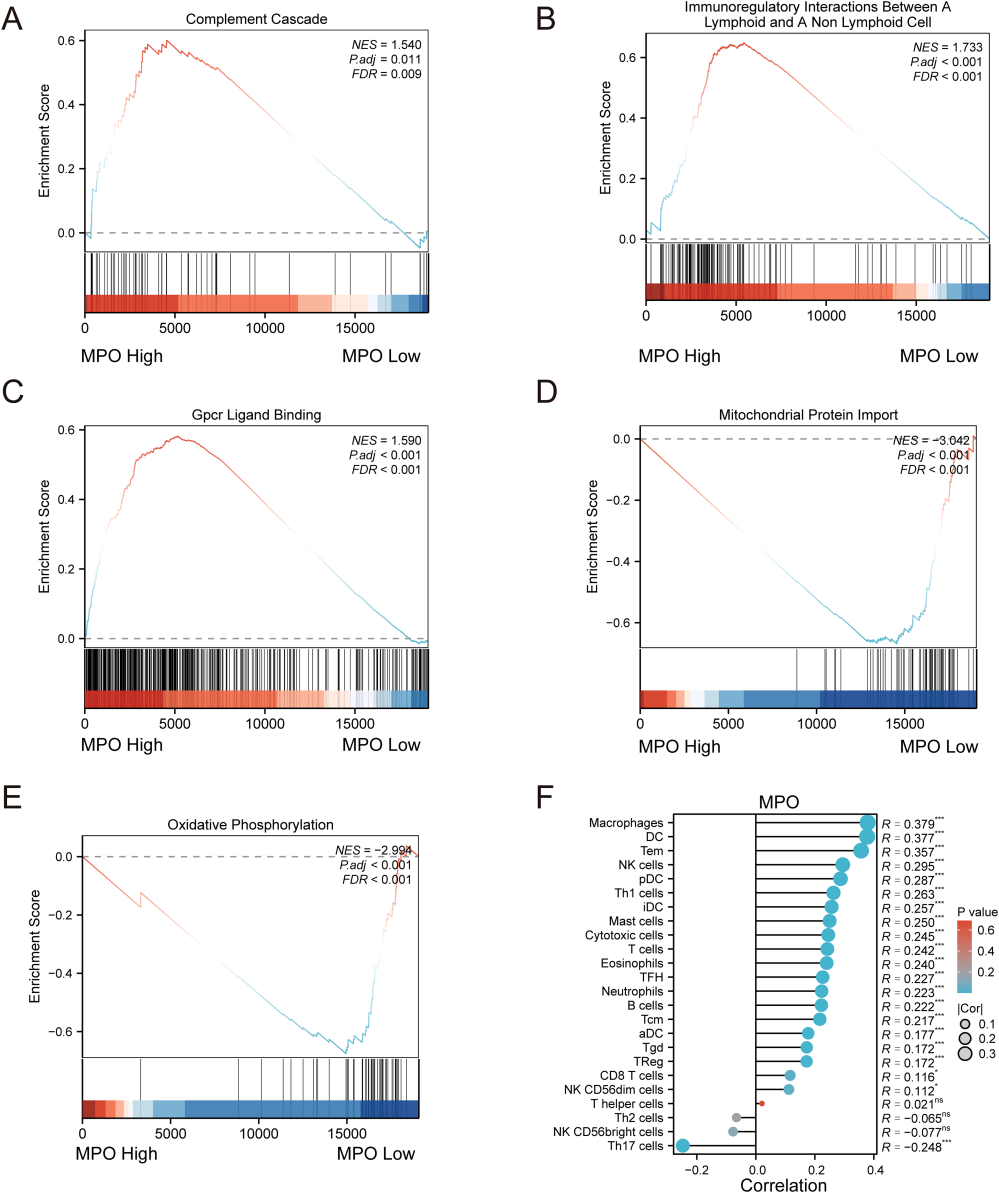
Gene set enrichment analysis (GSEA) and immune infiltration correlation analysis. **(A)** GSEA plot for Complement Cascade pathway, **(B)** Immunoregulatory Interactions Between a Lymphoid and a Non-Lymphoid Cell pathway, **(C)** GPCR Ligand Binding pathway, **(D)** Mitochondrial Protein Import pathway, and **(E)** Oxidative Phosphorylation pathway; **(F)** Lollipop plot showing correlation between MPO expression and immune cell infiltration.

Furthermore, Gene Set Enrichment Analysis (GSEA) revealed that high MPO expression was positively correlated with pathways such as Complement Cascade (**Figure 7A**), Immunoregulatory Interactions Between a Lymphoid and a Non-Lymphoid Cell (**Figure 7B**), and GPCR Ligand Binding (**Figure 7C**) (NES > 0), while low MPO expression was associated with Mitochondrial Protein Import (**Figure 7D**) and Oxidative Phosphorylation (**Figure 7E**) (NES < 0). These results suggest that MPO may play a role in immune regulation within the tumor microenvironment. To further investigate this, we performed single-sample gene set enrichment analysis (ssGSEA) to assess immune cell infiltration and correlated MPO expression with immune cell proportions. MPO expression showed significant positive correlations with various immune cell types, including B cells, cytotoxic T cells, regulatory T cells (Treg), natural killer cells (NK), dendritic cells (DC), and macrophages (all P < 0.05). Notably, the strongest correlations were observed with macrophages (R = 0.379, P < 0.001) and dendritic cells (R = 0.377, P < 0.001) (**Figure 7F**).

## Discussion

MPO can oxidize and modify DNA through its derived oxidants, thereby creating a high-mutagenic environment closely associated with tumorigenesis (12). In gastric cancer, patients or animal models exhibit features of increased MPO-positive cell infiltration and elevated expression levels, suggesting that MPO plays a critical role in gastric cancer (17, 22, 23). In this study, our goal was to investigate the relationship between MPO and pepsinogen levels, immune modulation, and prognosis in gastric cancer patients, to better understand MPO’s role in gastric health and cancer. Among the participants included in this study, the proportion of males was higher, which is consistent with previous epidemiological studies in China indicating that gastric cancer predominates in males (24). By dividing the study population into quartiles based on PGR, we found that participants in the low PGR group were older and had a higher Helicobacter pylori (H. pylori) infection rate, which aligns with the view that aging is a risk factor for gastric cancer (25). Furthermore, previous studies have shown that females have lower PGR compared to males, and individuals with H. pylori infection have lower serum PGR levels (26). It is well known that H. pylori infection significantly alters gastric pepsinogen levels (27), which supports our finding that the low PGR group had a higher rate of H. pylori infection. Although we also analyzed other factors, such as blood pressure and lipid levels, in relation to gastric pepsinogen, their effects appeared to be more indirect. Importantly, we found a significant inverse relationship between MPO levels and PGR, with higher MPO levels observed in the lower quartiles, suggesting a potential association between MPO and PGR. Therefore, in this study, we focus on the relationship between MPO and PGR.

To clarify the relationship between MPO and PGR, we employed a multiple regression model and found that, before adjusting for confounding factors, MPO was significantly negatively correlated with PGR and PGI, while showing a significant positive correlation with PGII. However, in Model II, after adjusting for factors such as gender, age, BMI, systolic blood pressure, and smoking status, PGII lost its correlation with MPO. These results suggest that the phenotypic changes induced by PGR may be related to variations in MPO levels. Notably, it has been reported that in a H. pylori-induced gastritis model, MPO activity exhibited an opposite trend to PGI levels (28), further suggesting that MPO may mediate PGI-associated gastric lesions. To further investigate the cellular sources of MPO and pepsinogens in gastric cancer, we performed single-cell RNA-seq analysis of gastric cancer (GSE210347) using scCancerExplorer (29). which revealed that MPO expression was predominantly localized to myeloid cells (particularly neutrophils), while PGI-related genes (PGA3/4/5) and PGII-associated gene (PGC) were exclusively expressed in normal gastric epithelial (**Supplementary Figure S6**). Previous studies have shown that neutrophils can promote gastric cancer progression by inducing epithelial-mesenchymal transition (EMT) (30). Therefore, these findings support an immune-epithelial crosstalk mechanism, whereby myeloid-derived MPO may regulate epithelial pepsinogen production through paracrine signaling, highlighting the interaction between immune infiltration and epithelial function in the gastric environment. Given that serum PGR is a biomarker for gastric cancer (18), to explore the potential link between MPO and gastric cancer, we conducted subgroup and interaction analyses while validating the relationship between MPO and PGR. We identified that the negative correlation between MPO and PGR was stronger in populations who smoked, drank alcohol, or had hypertension. Since smoking, alcohol consumption, and hypertension-related metabolic syndrome are risk factors for gastric cancer (31, 32), these findings suggest that the relationship between MPO and PGR is more pronounced in high-risk populations for gastric cancer. The results imply that MPO may be associated with gastric cancer progression.

To accurately describe the relationship between MPO and pepsinogen levels, we further investigated whether there was a linear relationship between MPO and pepsinogen levels. Using restricted cubic splines (RCS) and generalized additive models (GAM), we identified a threshold at an MPO value of 24 ng/ml. The results revealed a non-linear association between MPO and pepsinogen levels. Below the threshold, i.e., when MPO levels were less than 24 ng/ml, an increase in MPO was significantly associated with a decrease in PGR and PGI, while it was significantly correlated with an increase in PGII. This non-linear relationship suggests that the impact of MPO on pepsinogen levels may change within different MPO level ranges, providing a new perspective for understanding MPO’s role in physiological and pathological processes. Specifically, low MPO levels may be closely associated with decreased pepsinogen levels, whereas above the threshold of 24 ng/ml, the relationship between MPO and pepsinogen may weaken or become insignificant. Given that MPO is related to gastric mucosal damage (33), this could explain its impact on pepsinogen levels. However, further studies are needed to confirm whether the effects of MPO on gastric injury or pepsinogen vary across different MPO value ranges. Overall, the establishment of the threshold value of 24 ng/ml for MPO may provide a new reference biomarker for clinical use in assessing the relationship between MPO and changes in gastric pepsinogen levels.

This study explored the role of MPO in gastric cancer by analyzing data from 375 gastric adenocarcinoma patients in the TCGA cohort. The results indicated that high MPO expression was significantly associated with poor survival prognosis, particularly in disease-specific events, where high MPO expression was closely linked to disease progression in gastric adenocarcinoma. Furthermore, PFI and Kaplan-Meier survival analysis further supported the role of high MPO expression in promoting disease progression and poor survival outcomes. Multivariate analysis confirmed that MPO is an independent prognostic factor for overall survival and disease-specific survival. To assess the broader relevance of MPO, we performed Cox regression across 33 TCGA cancer types. While most cancers showed no significant association, gastrointestinal malignancies exhibited consistent adverse effects of high MPO expression: in gastric adenocarcinoma (STAD), high MPO predicted worse overall survival (OS: HR = 1.426, 95% CI 1.022–1.990, P = 0.037), disease-specific survival (DSS: HR = 1.667, 1.084–2.565, P = 0.020), and progression-free interval (PFI: HR = 1.631, 1.135–2.342, P = 0.008); similarly, colon adenocarcinoma (COAD) showed elevated risk for OS (HR = 1.588, 1.069–2.360, P = 0.022), DSS (HR = 2.032, 1.209–3.413, P = 0.008), and PFI (HR = 1.702, 1.192–2.429, P = 0.003) (data not shown). Previous studies have also highlighted the significant role of MPO in other tumors, such as its involvement in the immune-suppressive microenvironment of pancreatic cancer (14) and its potential biomarker value in ovarian cancer (34). Additionally, MPO has been shown to promote migration and invasion of human choriocarcinoma JEG-3 cells (15)., and inhibiting MPO can enhance the efficacy of melanoma immunotherapy (35). Therefore, MPO plays a potential therapeutic target role in various types of tumors.

Given that high MPO expression is associated with poor prognosis in gastric adenocarcinoma, MPO could potentially serve as a prognostic biomarker for gastric adenocarcinoma patients. Specifically, the detection of MPO levels can assist physicians in assessing the survival risk of patients, particularly in the early stages of treatment. Patients with high MPO expression may require more aggressive treatment regimens, including early intervention, close monitoring, and regular follow-ups. Although MPO is correlated with the malignancy and survival prognosis of gastric cancer, further functional experiments are needed to determine whether MPO holds potential as a therapeutic target for gastric cancer. To preliminarily explore the biological significance of MPO, we analyzed differentially expressed genes between the high and low MPO expression groups. The results showed that the differentially expressed genes were primarily associated with cell proliferation, invasion, G-protein-coupled receptors (GPCRs), and cAMP signaling pathways (36, 37). Importantly, GSEA results indicated that high MPO expression was positively correlated with complement cascade, immune regulation, and GPCR-related pathways, whereas low MPO expression was linked to mitochondrial protein import and oxidative phosphorylation. Previous studies in cancer research have shown that complement activation contributes to carcinogenesis (38), and aerobic glycolysis is crucial for cell survival in the tumor microenvironment (39, 40). These results suggest that MPO may play a role in the tumor microenvironment. Given that metabolic reprogramming of cancer cells and immune cells in the tumor microenvironment regulates the immune response to tumors (41), we aimed to assess the immune cell infiltration characteristics using ssGSEA. The results revealed that MPO exhibited strong correlations with macrophages and dendritic cells. Notably, tumor-associated macrophages play an important role in gastric cancer development (42). Additionally, dendritic cell subpopulations significantly affect immunity and tolerance in the cancer environment (43). Overall, these findings suggest that MPO may play a crucial role in tumor immune escape and progression by regulating immune cell infiltration and activity.

Our study has several key strengths. Firstly, we utilized data from a large sample size of 16,943 healthy individuals and 375 gastric adenocarcinoma patients, which enhances the reliability and generalizability of the findings. In particular, the TCGA cohort represents a broad and diverse patient population, providing a solid foundation for investigating the role of MPO in gastric adenocarcinoma. Secondly, the nonlinear relationship explored in this study and the identification of the 24 ng/ml threshold for MPO provides an important reference point for changes in gastric pepsinogen levels. Clinically, MPO levels may change during treatment, and regular monitoring of MPO levels can reflect the changes in gastric pepsinogen. Finally, this study is the first to comprehensively analyze the role of MPO in gastric health and gastric cancer, offering a potential new biomarker for the early diagnosis and prevention of gastric adenocarcinoma.

Despite these strengths, some limitations of our study should be acknowledged. First, the study primarily relied on cross-sectional data, which prevents us from establishing a causal relationship between MPO levels and gastric mucosal damage markers. Additionally, there is a lack of long-term follow-up data. Second, while we observed a correlation between MPO expression and gastric adenocarcinoma prognosis, the underlying mechanisms remain unclear, and further investigation is needed to explore MPO’s role in tumor immune escape and progression. Furthermore, the immune infiltration analysis was based solely on gene expression data, without validation through methods like immunohistochemistry, which may limit the robustness of the findings. Additionally, the participants in this study were all from healthy populations; extrapolation of the conclusion to other populations has certain limitations. We acknowledge that the inverse but non-linear MPO-PGR relationship observed in healthy cohorts represents a key limitation, as extrapolating these findings to disease states, such as gastric cancer, requires caution. Lastly, although we analyzed various subgroups (such as those with hypertension, smokers, and drinkers), the influence of factors like race and age was not explored in detail. Future research could consider population heterogeneity to assess the clinical applicability and biological significance of MPO across different subgroups. In conclusion, future studies should focus on the immune regulatory mechanisms of MPO in healthy populations, while emphasizing the need for further research in gastric adenocarcinoma cohorts. Specifically, the role of MPO in tumor immune escape and metastasis, through its modulation of immune cell infiltration and activity, warrants deeper investigation. Given its prognostic value, MPO’s potential as a therapeutic target, particularly in immunotherapy, should also be explored. Additionally, integrating multi-omics data for a more in-depth analysis will provide a more comprehensive understanding of MPO’s molecular mechanisms and facilitate its clinical application.

## Data Availability

The original contributions presented in the study are included in the article/**Supplementary Material**. Further inquiries can be directed to the corresponding authors.
