# Supplementary material for: Integrated analysis of myeloperoxidase in gastric health and cancer: associations with pepsinogen levels, immune regulation, and prognosis in a large healthy population-based and TCGA cohorts
Source: Front Immunol. 2025 Jun 6;16:1590257. doi: 10.3389/fimmu.2025.1590257 (PMC12179060; doi:10.3389/fimmu.2025.1590257)
Supplement: Supplementary file 1 [file DataSheet1.zip › supplementary material/supplementary material.docx]

***Supplementary Material***

**Integrated Analysis of Myeloperoxidase (MPO) in Gastric Health and Cancer: Associations with Pepsinogen Levels, Immune Regulation, and Prognosis in A Large Healthy Population-Based and TCGA Cohorts**

Junteng Zhou^1#^, Qihang Kong^2#^, Xiaojing Liu^2,3**^ and Yan Huang^1,4,5,6*^

^1^Health Management Center, General Practice Medical Center, West China Hospital, Sichuan University, Chengdu 610041, China

^2^Laboratory of Cardiovascular Diseases, Regenerative Medicine Research Center, West China Hospital, Sichuan University, Chengdu, China.

^3^Department of Cardiology, West China Hospital, Sichuan University, Chengdu, China

^4^State Key Laboratory of Respiratory Health and Multimorbidity

^5^Research Laboratory for Prediction and Evaluation of Chronic Diseases in the Elderly, National Clinical Research Center for Geriatric Diseases

^6^General Practice Research Institute, West China Hospital, Sichuan University, Chengdu, China.

*Correspondence to: Yan Huang, Health Management Center, General Practice Medical Center, West China Hospital, Sichuan University, Chengdu 610041, China.

E-mail: yanhuang@wchscu.cn;

**Xiaojing Liu, Laboratory of Cardiovascular Diseases, West China Hospital, Sichuan University, Chengdu, Sichuan Province, 610041, PR China.

E-mail: liuxq@scu.edu.cn.

#Junteng Zhou and Qihang Kong contributed equally to this work.


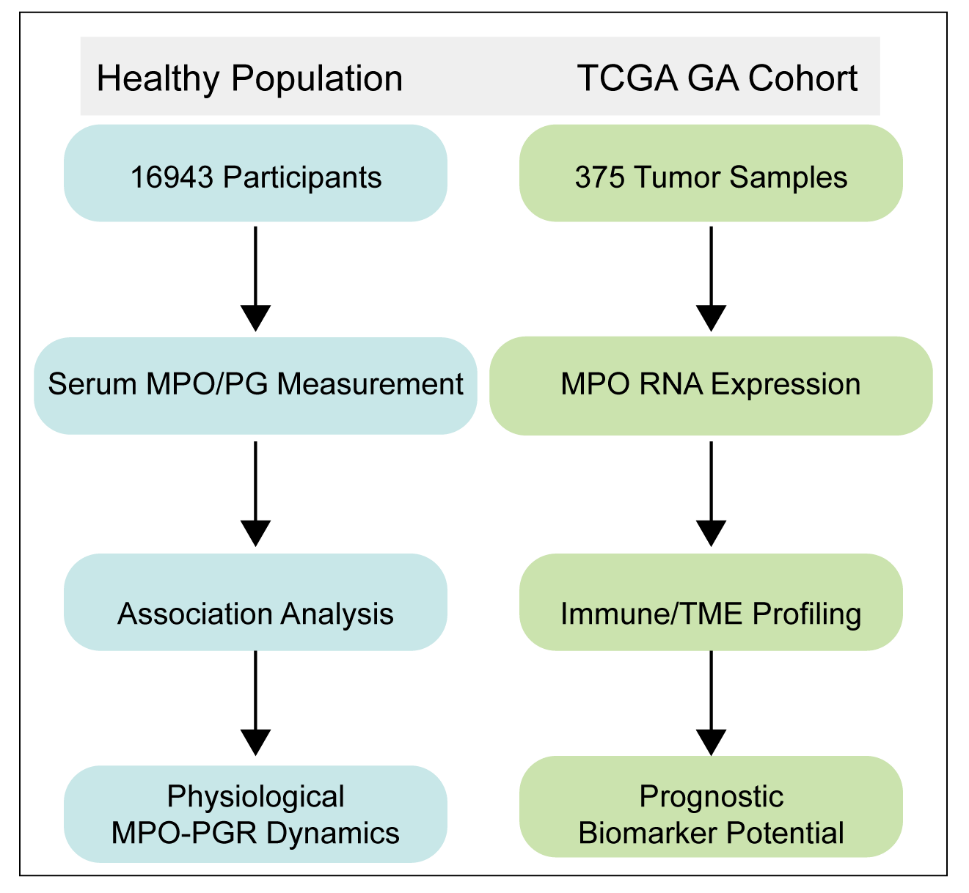


Figure S1. Overall study design.


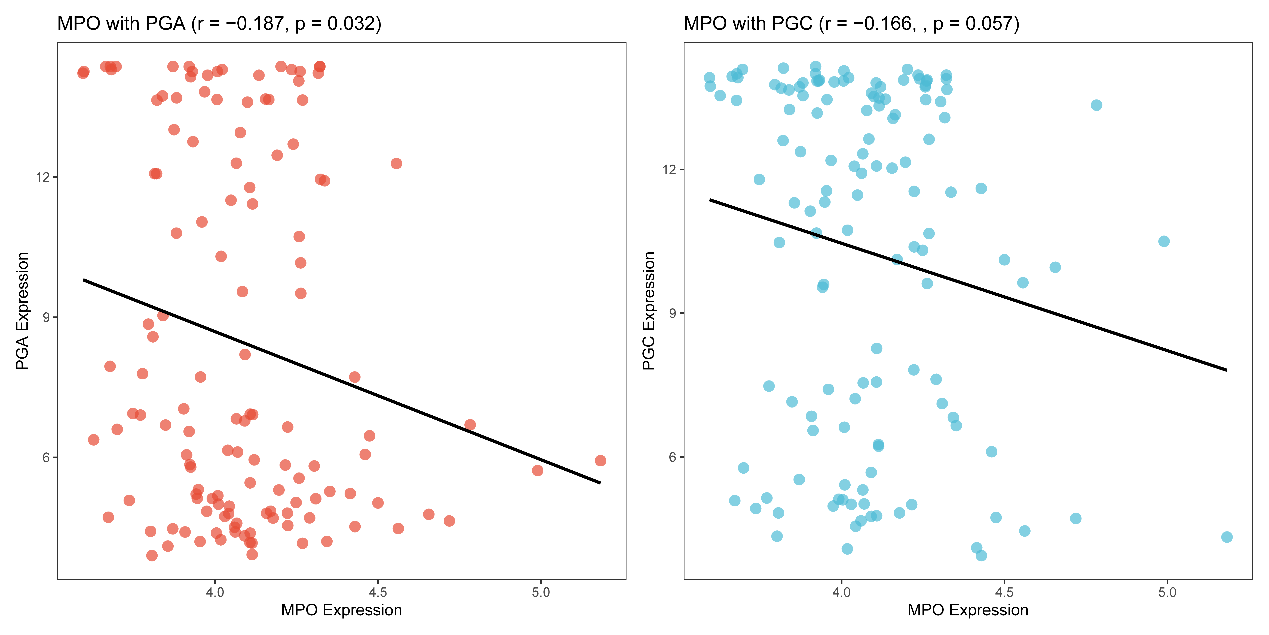


Figure S2. Correlation analysis of MPO expression with PGA and PGC expression.


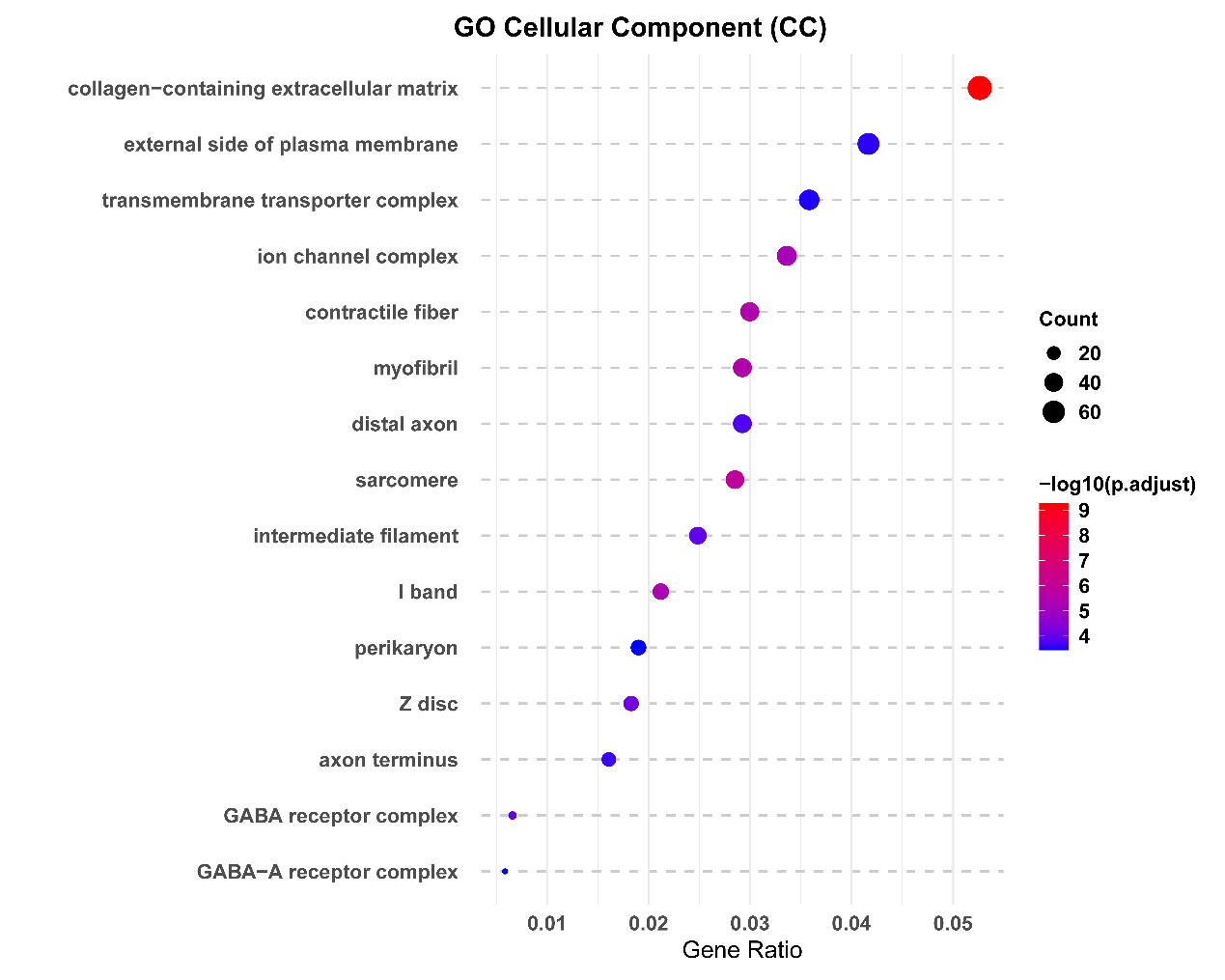


Figure S3. Cellular component (CC) enrichment analysis.

Bubble plot of enriched cellular components based on GO analysis.


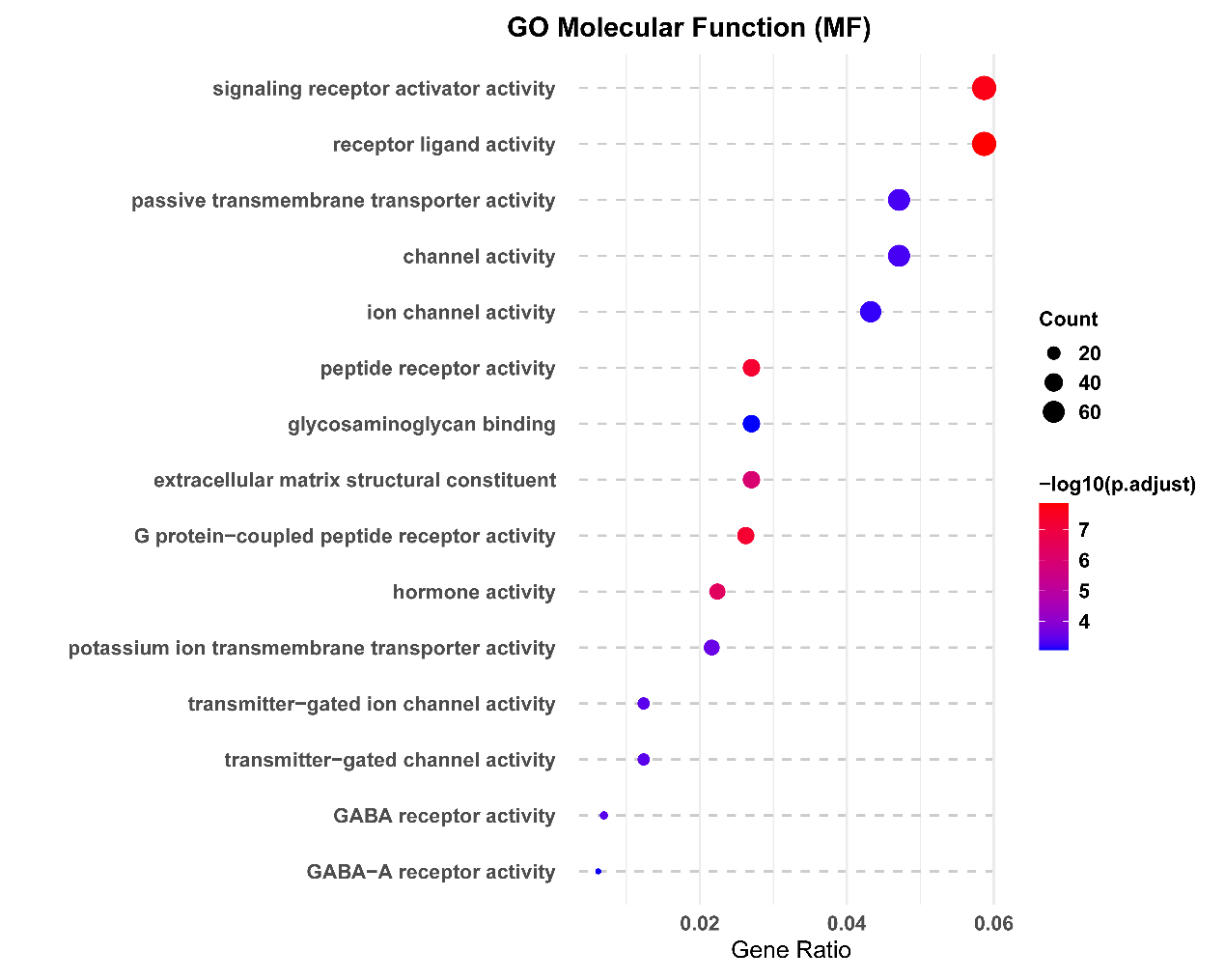


Figure S4. Molecular function (MF) enrichment analysis.

Bubble plot of enriched molecular functions based on GO analysis.


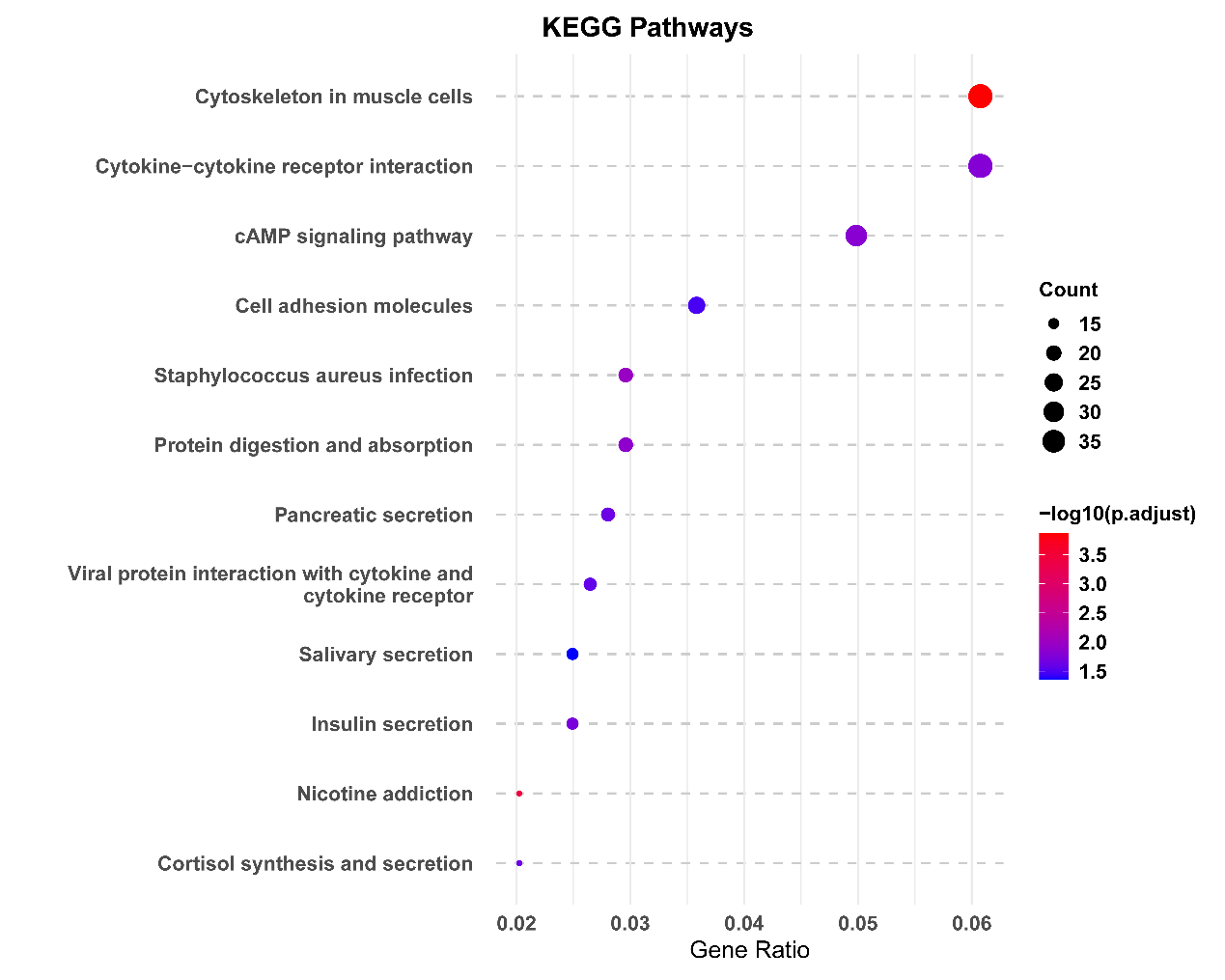


Figure S5. KEGG pathway enrichment analysis.

Bubble plot of enriched KEGG pathways.


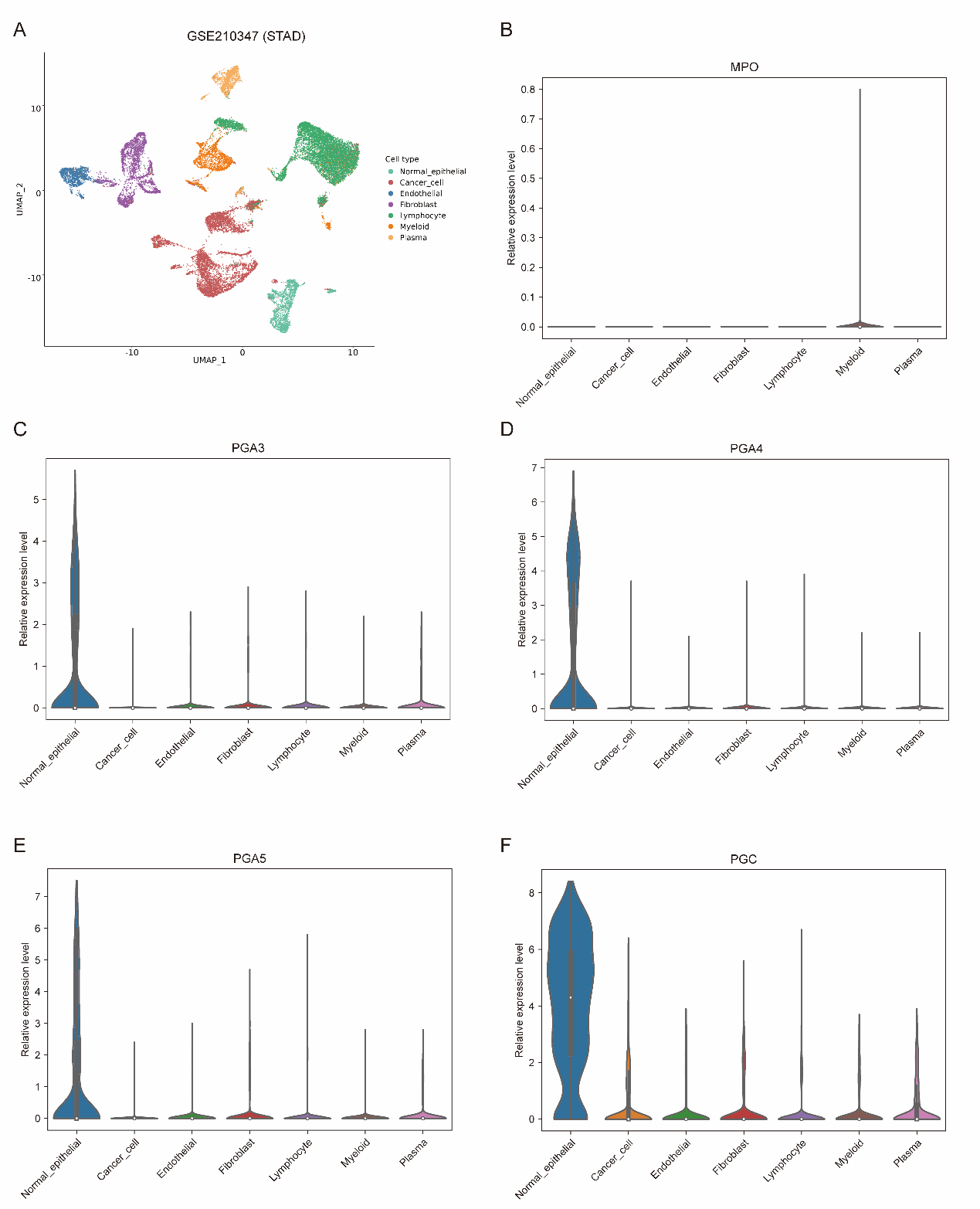


Figure S6. Single-cell transcriptomic data from GSE210347 were analyzed using scCancerExplorer. (A) UMAP plot of major cell types in gastric cancer tissues. (B) The distribution of MPO expression is predominantly localized to myeloid cells. (C-F) Violin plots comparing expression levels of PGI-related genes (PGA3/4/5) and PGII (PGC) across different cell subsets.

| Supplementary Table 1. Comparison of Baseline characteristics Across Clinically Defined PGR Groups (≤3 vs. >3) in the Healthy Population Cohort | | | |
| --- | --- | --- | --- |
|  |  |  |  |
|  | PGR <=3 | PGR >3 | P-value |
| No. of participants | 403 | 16540 |  |
| Age (years, mean ± SD) | 52.70 ± 12.38 | 45.44 ± 11.97 | <0.001 |
| Sex, N (%) |  |  | 0.008 |
| Female | 219 (54.34%) | 7892 (47.71%) |  |
| Male | 184 (45.66%) | 8648 (52.29%) |  |
| BMI (kg/m², mean ± SD) | 23.67 ± 3.19 | 23.49 ± 3.37 | 0.295 |
| SBP (mmHg, mean ± SD) | 119.64 ± 18.36 | 116.15 ± 15.79 | <0.001 |
| DBP (mmHg, mean ± SD) | 72.36 ± 11.10 | 71.84 ± 10.48 | 0.326 |
| Smoking status, N (%) |  |  | 0.002 |
| Never | 310 (76.92%) | 11995 (72.52%) |  |
| Former | 26 (6.45%) | 724 (4.38%) |  |
| Current | 67 (16.63%) | 3821 (23.10%) |  |
| Alcohol status, N (%) |  |  | 0.01 |
| Never | 248 (61.54%) | 9177 (55.48%) |  |
| Former | 6 (1.49%) | 128 (0.77%) |  |
| Current | 149 (36.97%) | 7235 (43.74%) |  |
| Hypertension, N (%) |  |  | <0.001 |
| No | 360 (89.33%) | 15468 (93.52%) |  |
| Yes | 43 (10.67%) | 1072 (6.48%) |  |
| Diabetes, N (%) |  |  | 0.129 |
| No | 389 (96.53%) | 16157 (97.68%) |  |
| Yes | 14 (3.47%) | 383 (2.32%) |  |
| Hyperlipidemia, N (%) |  |  | 0.375 |
| No | 399 (99.01%) | 16285 (98.46%) |  |
| Yes | 4 (0.99%) | 255 (1.54%) |  |
| Gout, N (%) |  |  | 0.968 |
| No | 398 (98.76%) | 16331 (98.74%) |  |
| Yes | 5 (1.24%) | 209 (1.26%) |  |
| RBC (*10^12/L, mean ± SD) | 4.77 ± 0.54 | 4.88 ± 0.54 | <0.001 |
| WBC (*10^9/L, mean ± SD) | 5.78 ± 1.47 | 5.84 ± 1.57 | 0.506 |
| Neutrophils (*10^9/L, mean ± SD) | 3.57 ± 1.23 | 3.49 ± 1.24 | 0.178 |
| Lymphocytes (*10^9/L, mean ± SD) | 30.41 ± 7.74 | 32.12 ± 7.47 | <0.001 |
| Monocytes (*10^9/L, mean ± SD) | 6.06 ± 1.67 | 6.02 ± 1.52 | 0.636 |
| Platelets (*10^9/L, mean ± SD) | 199.78 ± 61.23 | 206.09 ± 60.20 | 0.038 |
| TG (mmol/L, mean ± SD) | 1.40 ± 0.93 | 1.58 ± 1.29 | 0.007 |
| Cholesterol (mmol/L, mean ± SD) | 5.01 ± 0.99 | 4.94 ± 0.94 | 0.179 |
| LDL (mmol/L, mean ± SD) | 3.03 ± 0.82 | 2.96 ± 0.80 | 0.094 |
| HDL (mmol/L, mean ± SD) | 1.56 ± 0.44 | 1.51 ± 0.42 | 0.016 |
| ALT (U/L, median, IQR) | 19.00 (14.00-27.00) | 20.00 (14.00-29.00) | 0.344 |
| AST (U/L, median, IQR) | 22.00 (19.00-27.00) | 21.00 (17.00-25.00) | <0.001 |
| BUN (mmol/L, mean ± SD) | 5.07 ± 1.29 | 4.87 ± 1.24 | 0.001 |
| Creatinine (umol/L, mean ± SD) | 68.94 ± 15.23 | 70.12 ± 16.00 | 0.143 |
| MPO (ng/mL, median, IQR) | 27.71 (20.85-38.51) | 25.04 (18.23-35.27) | <0.001 |
| Pepsinogen I (ng/ml, median, IQR) | 45.79 (20.91-79.57) | 69.65 (55.14-90.57) | <0.001 |
| Pepsinogen II (ng/ml, median, IQR) | 20.97 (13.29-33.80) | 8.67 (6.46-12.44) | <0.001 |
| Pepsinogen ratio | 2.06 ± 0.71 | 8.21 ± 2.79 | <0.001 |
| H. pylori infection, N (%) |  |  | <0.001 |
| No | 158 (44.13%) | 10343 (69.65%) |  |
| Yes | 200 (55.87%) | 4508 (30.35%) |  |

Supplementary Table 2. Multivariate analysis of MPO’s association with PGR ≤ 3

| **Pepsinogen ratio <= 3** | Non-adjusted | Adjust model I | Adjust model II |
| --- | --- | --- | --- |
| MPO as Continuous | 1.003 (1.000, 1.006) 0.04843 | 1.003 (1.001, 1.006) 0.01551 | 1.003 (1.000, 1.006) 0.03298 |
| MPO as quartile |  |  |  |
| Q1 | Ref | Ref | Ref |
| Q2 | 1.199 (0.874, 1.644) 0.26080 | 1.198 (0.872, 1.645) 0.26483 | 1.212 (0.867, 1.696) 0.26110 |
| Q3 | 1.686 (1.255, 2.265) 0.00053 | 1.726 (1.283, 2.322) 0.00031 | 1.751 (1.277, 2.400) 0.00050 |
| Q4 | 1.758 (1.312, 2.357) 0.00016 | 1.936 (1.442, 2.600) 0.00001 | 1.989 (1.448, 2.731) 0.00002 |
| P for trend | 0.00002 | <0.00001 | <0.00001 |

Non-adjusted model: No adjustments.

Adjusted model I: Adjusted for sex and age.

Adjusted model II: Adjusted for sex, age, BMI, systolic blood pressure, smoking status, alcohol consumption, diabetes, gout, H. pylori infection, red blood cell count, white blood cell count, neutrophil count, lymphocyte count, platelet count, triglycerides, cholesterol, low-density lipoprotein, high-density lipoprotein, aspartate aminotransferase, and creatinine.

β-values : Unstandardized regression coefficients. 95% CI : 95% confidence interval.

| Supplementary Table 3. Association Between MPO and Pepsinogen Ratio (PGR) Stratified by H. pylori Infection Status in Multivariable Linear Regression Models | | |
| --- | --- | --- |
|  |  |  |
|  | H. pylori negative | H. pylori positive |
| **Non-adjusted** |  |  |
| MPO as Continuous | -0.008 (-0.010, -0.006) <0.00001 | -0.008 (-0.010, -0.005) <0.00001 |
| MPO as quartile |  |  |
| Q1 | Ref | Ref |
| Q2 | -0.745 (-0.896, -0.595) <0.00001 | -0.436 (-0.650, -0.221) 0.00007 |
| Q3 | -1.033 (-1.183, -0.883) <0.00001 | -0.673 (-0.887, -0.459) <0.00001 |
| Q4 | -0.875 (-1.028, -0.723) <0.00001 | -0.686 (-0.894, -0.479) <0.00001 |
| P for trend | <0.0001 | <0.0001 |
|  |  |  |
| **Adjust model I** |  |  |
| MPO as Continuous | -0.009 (-0.010, -0.007) <0.00001 | -0.009 (-0.011, -0.006) <0.00001 |
| MPO as quartile |  |  |
| Q1 | Ref | Ref |
| Q2 | -0.752 (-0.900, -0.603) <0.00001 | -0.442 (-0.651, -0.233) 0.00003 |
| Q3 | -1.044 (-1.193, -0.896) <0.00001 | -0.711 (-0.920, -0.502) <0.00001 |
| Q4 | -0.943 (-1.094, -0.792) <0.00001 | -0.784 (-0.988, -0.580) <0.00001 |
| P for trend | <0.0001 | <0.0001 |
|  |  |  |
| **Adjust model II** |  |  |
| MPO as Continuous | -0.009 (-0.011, -0.007) <0.00001 | -0.008 (-0.011, -0.005) <0.00001 |
| MPO as quartile |  |  |
| Q1 | Ref | Ref |
| Q2 | -0.759 (-0.907, -0.611) <0.00001 | -0.447 (-0.656, -0.237) 0.00003 |
| Q3 | -1.028 (-1.177, -0.880) <0.00001 | -0.695 (-0.907, -0.482) <0.00001 |
| Q4 | -0.967 (-1.121, -0.812) <0.00001 | -0.755 (-0.966, -0.543) <0.00001 |
| P for trend | <0.0001 | <0.0001 |

Non-adjusted model: No adjustments.

Adjusted model I: Adjusted for sex and age.

Adjusted model II: Adjusted for sex, age, BMI, systolic blood pressure, smoking status, alcohol consumption, diabetes, gout, red blood cell count, white blood cell count, neutrophil count, lymphocyte count, platelet count, triglycerides, cholesterol, low-density lipoprotein, high-density lipoprotein, aspartate aminotransferase, and creatinine.

β-values : Unstandardized regression coefficients. 95% CI : 95% confidence interval.

Supplementary Table 4. Univariate and multivariate analysis of MPO with OS

| Characteristics | Total(N) | HR(95% CI) Univariate analysis | P value Univariate analysis | HR(95% CI) Multivariate analysis | P value Multivariate analysis |
| --- | --- | --- | --- | --- | --- |
| Age | 367 |  |  |  |  |
| <= 65 | 163 | Reference |  | Reference |  |
| > 65 | 204 | 1.620 (1.154 - 2.276) | 0.005 | 1.858 (1.291 - 2.675) | < 0.001 |
| Gender | 370 |  |  |  |  |
| Female | 133 | Reference |  |  |  |
| Male | 237 | 1.267 (0.891 - 1.804) | 0.188 |  |  |
| Pathologic T stage | 362 |  |  |  |  |
| T1&T2 | 96 | Reference |  | Reference |  |
| T3&T4 | 266 | 1.719 (1.131 - 2.612) | 0.011 | 1.407 (0.893 - 2.217) | 0.141 |
| Pathologic N stage | 352 |  |  |  |  |
| N0&N1 | 204 | Reference |  | Reference |  |
| N2&N3 | 148 | 1.650 (1.182 - 2.302) | 0.003 | 1.668 (1.173 - 2.373) | 0.004 |
| H pylori infection | 162 |  |  |  |  |
| No | 144 | Reference |  |  |  |
| Yes | 18 | 0.650 (0.279 - 1.513) | 0.317 |  |  |
| Pathologic M stage | 352 |  |  |  |  |
| M0 | 327 | Reference |  | Reference |  |
| M1 | 25 | 2.254 (1.295 - 3.924) | 0.004 | 2.736 (1.527 - 4.904) | < 0.001 |
| MPO | 370 | 1.238 (1.062 - 1.443) | 0.006 | 2.781 (1.463 - 5.285) | 0.002 |

Supplementary Table 5. Univariate and multivariate analysis of MPO with DSS

| Characteristics | Total(N) | HR(95% CI) Univariate analysis | P value Univariate analysis | HR(95% CI) Multivariate analysis | P value Multivariate analysis |
| --- | --- | --- | --- | --- | --- |
| Age | 346 |  |  |  |  |
| <= 65 | 160 | Reference |  |  |  |
| > 65 | 186 | 1.211 (0.797 - 1.840) | 0.371 |  |  |
| Gender | 349 |  |  |  |  |
| Female | 125 | Reference |  | Reference |  |
| Male | 224 | 1.573 (0.985 - 2.514) | 0.058 | 1.588 (0.974 - 2.588) | 0.064 |
| Pathologic T stage | 345 |  |  |  |  |
| T1&T2 | 90 | Reference |  | Reference |  |
| T3&T4 | 255 | 2.089 (1.192 - 3.660) | 0.010 | 1.642 (0.903 - 2.986) | 0.104 |
| Pathologic N stage | 334 |  |  |  |  |
| N0&N1 | 192 | Reference |  | Reference |  |
| N2&N3 | 142 | 2.110 (1.378 - 3.231) | < 0.001 | 2.000 (1.282 - 3.120) | 0.002 |
| H pylori infection | 157 |  |  |  |  |
| No | 139 | Reference |  |  |  |
| Yes | 18 | 0.558 (0.200 - 1.554) | 0.264 |  |  |
| Pathologic M stage | 333 |  |  |  |  |
| M0 | 311 | Reference |  | Reference |  |
| M1 | 22 | 2.438 (1.221 - 4.870) | 0.012 | 2.351 (1.164 - 4.749) | 0.017 |
| MPO | 349 | 3.324 (1.651 - 6.692) | < 0.001 | 3.667 (1.704 - 7.890) | < 0.001 |

Supplementary Table 6. Univariate and multivariate analysis of MPO with PFI

| Characteristics | Total(N) | HR(95% CI) Univariate analysis | P value Univariate analysis | HR(95% CI) Multivariate analysis | P value Multivariate analysis |
| --- | --- | --- | --- | --- | --- |
| Age | 369 |  |  |  |  |
| <= 65 | 164 | Reference |  |  |  |
| > 65 | 205 | 0.858 (0.603 - 1.221) | 0.395 |  |  |
| Gender | 372 |  |  |  |  |
| Female | 133 | Reference |  | Reference |  |
| Male | 239 | 1.638 (1.099 - 2.440) | 0.015 | 2.505 (1.355 - 4.631) | 0.003 |
| Pathologic T stage | 364 |  |  |  |  |
| T1&T2 | 97 | Reference |  | Reference |  |
| T3&T4 | 267 | 1.705 (1.095 - 2.654) | 0.018 | 1.238 (0.637 - 2.407) | 0.529 |
| Pathologic N stage | 354 |  |  |  |  |
| N0&N1 | 205 | Reference |  | Reference |  |
| N2&N3 | 149 | 1.892 (1.325 - 2.703) | < 0.001 | 2.134 (1.248 - 3.650) | 0.006 |
| H pylori infection | 163 |  |  |  |  |
| No | 145 | Reference |  | Reference |  |
| Yes | 18 | 0.321 (0.100 - 1.024) | 0.055 | 0.338 (0.104 - 1.105) | 0.073 |
| Pathologic M stage | 353 |  |  |  |  |
| M0 | 328 | Reference |  | Reference |  |
| M1 | 25 | 2.224 (1.194 - 4.144) | 0.012 | 2.242 (0.923 - 5.448) | 0.075 |
| MPO | 372 | 1.159 (0.952 - 1.411) | 0.142 |  |  |
